# Ribonucleoprotein particles of bacterial small non-coding RNA IsrA (IS61 or McaS) and its interaction with RNA polymerase core may link transcription to mRNA fate

**DOI:** 10.1093/nar/gkv1302

**Published:** 2015-11-24

**Authors:** Rob W. van Nues, Daniel Castro-Roa, Yulia Yuzenkova, Nikolay Zenkin

**Affiliations:** Centre for Bacterial Cell Biology, Institute for Cell and Molecular Biosciences, Newcastle University, Newcastle upon Tyne, NE2 4AX, UK

## Abstract

Coupled transcription and translation in bacteria are tightly regulated. Some small RNAs (sRNAs) control aspects of this coupling by modifying ribosome access or inducing degradation of the message. Here, we show that sRNA IsrA (IS61 or McaS) specifically associates with core enzyme of RNAP *in vivo* and *in vitro*, independently of σ factor and away from the main nucleic-acids-binding channel of RNAP. We also show that, in the cells, IsrA exists as ribonucleoprotein particles (sRNPs), which involve a defined set of proteins including Hfq, S1, CsrA, ProQ and PNPase. Our findings suggest that IsrA might be directly involved in transcription or can participate in regulation of gene expression by delivering proteins associated with it to target mRNAs through its interactions with transcribing RNAP and through regions of sequence-complementarity with the target. In this eukaryotic-like model only in the context of a complex with its target, IsrA and its associated proteins become active. In this manner, in the form of sRNPs, bacterial sRNAs could regulate a number of targets with various outcomes, depending on the set of associated proteins.

## INTRODUCTION

The discovery of small non-coding RNAs in bacteria (sRNAs) that regulate translation and/or levels of particular mRNAs by base-pair interactions together with proteins such as the general RNA chaperone Hfq (1–3) is both as exciting as the multitude of involved mechanisms is puzzling (4). Translational regulation by sRNAs is often modeled as an interference with ribosome binding by occlusion of the ribosome binding site or the start codon just downstream. Alternatively, sRNAs appear to induce degradation of the message (1) but the exact mechanisms by which this occurs are fairly speculative (4). In eukaryotes, small non-coding RNAs also participate in many steps required in the control of gene expression. In the form of ribonucleoprotein particles (RNPs) they assemble, for example, on precursor-mRNAs (pre-mRNAs) or on ribosomal RNA precursors (pre-rRNAs) in large complexes for the removal of introns or external and internal transcribed spacers (in the case of pre-rRNA) and post-transcriptional modification. These small RNAs contain sequences that bind (a set of) core proteins essential for the stability or function of the RNP, guide the RNP to their target as well as take part in catalysis. Target RNA maturation steps are in part co-transcriptional and, in the case of splicing, organized by factors associating with the C terminal domain of RNA polymerase (RNAP) (5–7).

Many of known sRNAs in bacteria regulate translation by interacting with sequences of the mRNA and so modulate ribosome binding (1–3). In bacteria, on many genes, translation is coupled to transcription (8–10), i.e. the ribosome binds the nascent mRNA right behind the transcribing RNAP. Recent study revealed that RNAP pauses in the vicinity of the translation start site (11), possibly to ensure proper coupling. This suggests a possibility that regulation of translation by sRNAs may also take place in a co-transcriptional manner and perhaps involve interaction of sRNA with RNAP. Indeed, RNAP has been reported to interact with a number of sRNAs and proteins known to associate with them, such as Hfq or protein S1, a component of the small ribosomal subunit (12–14).

The best-studied interaction of bacterial RNAP with a non-coding RNA is that with 6S RNA, an 183 nt RNA that binds the RNAP holo-enzyme by mimicking an open transcription bubble, and, thus, regulates initiation of transcription. 6S RNA accumulates when nutritional sources begin to run out and cellular growth becomes stationary. During exit from stationary phase, 6S RNA serves as a template for RNAP and by generation of product RNA the polymerase gets released and becomes available for transcription (15). Non-coding RNAs that regulate the elongating RNAP are known only in eukaryotes. These include the U1 snRNA, best known for its involvement in the formation of splicing complexes (16), and the 7SK snRNA, which controls pausing of RNAP downstream of the transcription start sites of particular genes during transcript elongation and contributes to termination (17–19).

In this work, we sought regulatory RNAs that would associate with RNAP. We identified a small non-coding RNA that specifically interacts with core enzyme of RNAP at a site distinct from the main nucleic acids binding cleft, thus pointing to its possible role in transcription elongation. This sRNA, IsrA, originally identified (but imprecisely) in a bioinformatic screen for novel transcription units on the chromosome as IS61 (20), has been found to interfere with the expression of genes like *csgD* or *flhDC*, involved in the multi-cellular adhesive lifestyle of *E. coli* and was renamed to McaS (21,22). As, however, network analysis and subsequent mutational analysis had implicated IsrA in DNA repair (23) and not only in processes regulating motility or biofilm formation (21,22), we prefer to use the original, more general name for this sRNA. In addition to RNAP core, Hfq, protein S1, CsrA, ProQ and PNPase, were also found to associate with IsrA *in vivo*, suggesting that IsrA may regulate translation as an RNP and possibly do so in a co-transcriptional manner.

## MATERIALS AND METHODS

### Construction of strains and IsrA mutants

Parental and recombinant strains obtained as described below are listed in the Supplementary Table. PCR reactions were done with Phusion DNA polymerase (Finnzymes) and primers from IDT. All gene-replacement constructs resulted from ligation of a PCR-amplified, kinased fragment containing the selectable marker and a gapped vector-fragment obtained by inverse PCR of the plasmid in which the target gene had been cloned (see below). DNA templates were removed by DpnI (NEB) digestion.

#### Strain with biotinylated RpoC

For one-step purification of RNAP with streptavidin sepharose (GE Healthcare) the biological active C-terminus of AccB, the biotin carboxyl carrier protein (BCCP) (aa 71–156) (24)) was fused to *rpoC* by means of a linker containing the cleavage site for human rhino virus 3C (HRV3C) protease (LEVLFQ/GP). Multiple rounds of Exponential megapriming PCR (25) yielded an integration cassette based on plasmid pAMD001 (26) in which the *thyA* gene was replaced by the kanamycin resistance marker from pKD4 (27) that was amplified (using primers 5′-TTCAAGATCCCCTCACGCTG and 5′-TTCAGAGCGCTTTTGAAGCTG) without the FRT sequences (Supplementary Figure S1). The BCCP-tagging cassette was amplified (with primers 5′-CGATATCGAAAGAGAACGTTATCGTG and 5′-CATCCAACGCTCTAGATCTGTC), treated with DpnI, and integrated into the *rpoC* locus of strain BW25113 using the λRed recombinase system (27). P1 lysates prepared from kanamycin resistant recombinant cells with the correct genomic insertions (as confirmed by colony PCR), were used for horizontal transfer of *accB*-tagged *rpoC* to other strains (Supplementary table).

#### Disruption of the chromosomal loci for Hfq and IsrA

pGemT Easy (Promega) plasmids were constructed containing the *hfq* ORF, which was amplified by PCR with 102 bp upstream and 84 bp downstream sequence from MG1655, or the IsrA coding region including 394 bp upstream and 233 bp downstream sequence, amplified from MG1655, JC7623 or RL*^rpoC^*^HIS^, which yielded pThfq or pTisrA-M, −J or −R, respectively) (Supplementary Figure S1D and S1E). In these plasmids, the coding regions 196–282 or 16–282 of *hfq* and 3–56 of *isrA* were replaced with the chloramphenicol resistance gene from pKD3 (amplified with primers annealing to sites p1 and p2) (27) to obtain integration cassettes for maintaining the 65 aa core of Hfq (hfq65) or for complete disruption of *hfq* and *isrA* genes. Integration of the cassettes by means of λRed recombinase (27) was verified by colony PCR on chloramphenicol resistant cells with, in the case of *hfq* disruption, an upstream outer primer and c1 (27) or with up- and downstream outer primers, and, in the case of *isrA* disruption, with c1 and an upstream outer primer as shown in Supplementary Figure S1 (panels D and E).

#### Strains carrying IsrA-mutants

Mutants of IsrA (shown in Supplementary Figure S1F) were prepared by inverse PCR of pTisrA-R. The plasmids were tested *in vivo* after transformation of strains lacking endogenous IsrA or used as templates for the production of T7 templates for *in vitro* RNA synthesis.

### Purification of RNPs through RNAP

#### Culturing of cells

Cells were grown on LB (10 g/l tryptone, 5 g/l yeast extract, 10 g/l NaCl) with antibiotics (100 μg/ml ampicillin, 50 μg/ml kanamycin or 25 μg/ml chloroamphenicol) as needed. For co-immunoprecipitation experiments with His_6_-tagged RpoC cells were grown to mid-exponential, early—or late stationary phase, washed with water and stored at −80°C. For co-immunoprecipitation experiments with BCCP-tagged RpoC, cells were cultured in the presence of 100 mg/l biotin (Sigma). Overnight grown pre-cultures were diluted 100-fold into 150 ml medium and grown to an OD600 of ∼1.6–1.9, after which the cells were harvested by centrifugation at 4°C, washed three times with 50 ml cold PBS (8 g/l NaCl, 0.2 g/l KCl, 1.8 g/l Na_2_HPO_4_·2 H_2_O, 0.27 g/l KH_2_PO_4_) and stored at −80°C.

#### Purification of RNAP on nickel - or streptavidin sepharose

Cell pellets were resuspended on ice in 15–20 ml binding buffer (BB, 5% glycerol, 10 mM Tris–HCl pH 7.8, 1 mM MgCl_2_ and, for binding to streptavidin sepharose, 1 mM DTT) + 200 mM KCl (BB @ 200 mM KCl), 10 μl Superasin (Ambion), and EDTA-free Complete Protease Inhibitor (Roche) and broken by four rounds of 1 min 30 s sonication (power 30%, 1.5 s pulse on, 0.5 s off). Cell-debris was removed by centrifugation (2 × 10 min 7K rpm, 20 min 18K rpm) and the lysates were filtered over Millex-HV 0.45 μm PVDF filter-units (Millipore) before mixing with Ni Sepharose 6 Fast Flow or Streptavidin Sepharose High performance (GE Healthcare) beads. Extracts were incubated for 2–16 h at 4°C on a rotating wheel. To remove cellular biotin and endogenous BCCP, streptavidin sepharose beads were replaced after a pre-incubation period of 15–20 min. Beads were washed extensively with BB @ 200 mM KCl (including 20 mM imidazole, pH 7.8 when RNAP was bound to nickel sepharose), incubated with RNase-free DNAse (Roche) for 20–40 min to remove bound DNA, and washed with BB @ 200 mM KCl + 100 μg/ml heparin (Sigma) to release non-specifically bound RNA. RNAP was eluted from streptavidin sepharose by incubation with HRV3C (Sigma) for 16 h at 4°C in BB @150mM NaCl (in the presence of Superasin) and removed from nickel sepharose by digestion with proteinase K (Fluka). RNA was isolated from eluates by phenol–chloroform (Sigma), and chloroform (Sigma) extractions followed by ethanol precipitation in the presence of glycogen (Roche) as a carrier. Alternatively, RNAs were directly extracted from beads without enzymatic elution. RNAs were dissolved in 15 μl 15 mM Tris–HCl, pH 7.8 and stored at −20°C. Proteins were precipitated from the phenol-phase with acetone and dissolved in 15–30 μl 10 M urea. As an input control, total RNA was isolated from 100 μl extract and stored in 75 μl 15 mM Tris–HCl, pH 7.8.

#### RNA identification

Of a 200 μM stock, 0.33 μl of a mixed ribo-deoxyoligonucleotide with blocked 3′-end ([5Phos]rCrUrGGTAAGTCGACGCGTATCAAG-3ddC; IDT) was ligated in a 5 μl reaction to 2 μl of the RNA co-purified with RNAP in the presence of 0.33 μl (6 u) Superasin and 0.33 μl (3 u) T4 RNA ligase (ssRNA ligase, NEB) for 16 h at 15°C. RNA was purified from the reaction by phenol–chloroform extraction and ethanol precipitated in the presence of 0.3 M NaOAc, pH 5.2, and dissolved in 6 μl H_2_O. In an end-volume of 5 μl, 2 μl 3′-tagged RNA was annealed to 0.5 μl 100 μM RT-PCR primer (5′-TTGATACGCGTCGACTTACCAG) using a PCR machine (5 min 70°C, 5 min 4°C) and cDNA was prepared at 50°C for 45–60 min in a 10 μl reaction with 0.25 μl (50 u) Superscript III (Invitrogen) or Revertaid (Fermentas) reverse transcriptase, 2 μl 5× RT buffer (250 mM Tris–HCl, pH 8.3, 30 mM MgCl_2_, 375 mM KCl, 50 mM DTT), 1 μl 10 mM dNTPs (GE Healthcare), 0.5 μl (10 u) Superasin. The RT reaction included 0.5 μl 100 μM ‘Smart ligation primer’ (5′-ATCAATGTACGCGTCGACCrArGrGrG) to enrich for full-length products as described in the user manual for the SMART cDNA Library Construction Kit (Clontech). Alternatively, the 5′-adapter was ligated to purified cDNA in a separate reaction. Excess primer was removed by adding 0.5 μl (10 u) exonuclease I (NEB) and 1.1 μl 10× exonuclease I reaction buffer followed by a 30 min digestion at 37°C. Synthesized cDNA was purified as described above for 3′-end tagged RNA, taken up in 10 μl H_2_O, and 2 μl was amplified by 26–31 cycles of PCR with Phusion DNA polymerase (Finnzymes), the RT-PCR primer and a primer recognizing the 5′-adapter (5′-ATCAATGTACGCGTCGACCAG). PCR products in the range of 50–200 bp were gel-purified, kinased and cloned into pGemT Easy (Promega) that was cut with Ecl136 II (Fermentas) and treated with CIP (NEB). Clones with an insert as found by MluI (Promega) digestion were sequenced (by GATC-Biotech).

### RNA analysis

#### Northern blotting

RNAs (4 μg total RNA or 2 μl of the RNA co-purified with RNAP) separated on 10% polyacryl amide with 8M urea in TBE (10.9 g/l Tris, 5.6 g/l boric acid, 0.74 g EDTA) were blotted onto Hybond NX (GE Healthcare) in 0.5× TBE using a Trans-blot Cell with plate electrodes (NEB), crosslinked with UV and prehybridized in SES1 (250 mM sodium phosphate pH 7.2, 7% SDS, 1 mM EDTA). Blots were hybridized overnight at 30°C in SES1 containing oligos against IsrA nt 4–26 or 6S nt 79–97 (of the mature, processed sRNA), which had been labeled with T4 kinase (NEB) and [γ-^32^P]-ATP (6000 Ci/mmol; Hartmann), and purified on G50 Micro Spin Columns (GE Healthcare). Blots were washed three times 15 min in SES1, exposed to a phosphor-imaging plate and analyzed using ImageQuant software (GE Healthcare).

#### End labeling

In 4 μl reactions, total RNA (0.5–1 μg), 2 μl of the RNA co-purified with RNAP or *in vitro* synthesized RNA (0.2 μg) were 3′ end labeled with 0.2 μl [5′-^32^P]-PcP (3000 Ci/mmol; Hartmann), 0.4 μl 10× T4 RNA ligase buffer, 0.4 μl DMSO, 0.3 μl T4 RNA ligase (NEB, Fermentas) and 0.2 μl Superasin for 1–1.5 h at 37°C. Likewise, the 5′ ends of RNAs were labeled with T4 kinase (NEB), 10× T4 kinase buffer and [у-^32^P]-ATP (6000 Ci/mmol; Hartmann) in the presence of Superasin. Labeled RNAs were separated on 10% polyacrylamide with 8M urea in TBE and fixed with 50% methanol, 10% acetic acid. Dried gels were exposed to a phosphor-imaging plate and analyzed using ImageQuant software.

### *In vitro* assays

Preparations of σ^70^, RNAP (core) enzyme (isolated from RL*^rpoC^*^HIS^ or MG1655*^rpoC^*^HIS^ that were kindly provided by Robert Landick and Rachel Mooney; Supplementary Table), and T7 RNAP transcribed RNAs (radioactively labeled with either [α-^32^P]-GTP, [5′-^32^P]-PcP or [γ-^32^P]-ATP and gel-purified) were as described previously (28). Mass-spectrometry (MS/MS) analysis of the RNAP preparations was done to check for contamination with proteins such as Hfq.

#### Electrophoretic mobility shift assays

EMSA in Figure 3A was done as described previously (29). Note that no yeast RNA was added before complex formation as in the EMSA experiments below.

EMSAs in Figures 3B and 4B, 4C were performed using procedures adapted from (30,31). Before complex formation in the case of Figures 3B, 4B and C, for each sample, 0.5 pmol *in vitro* synthesized radioactively labeled substrate RNA and, where indicated, 7–8 pmol specific competitor RNA, were denatured in 15 mM Tris–HCl pH 8 at 65°C for 5 min and refolded at 37°C for 10 min after adding 1 vol. 2× EMSA buffer (30 mM Tris–HCl pH8, 200 mM KCl, 2 mM MgCl_2_, 2 mM DTT) and put on ice. As a nonspecific competitor, 1 μg purified total yeast RNA in 1× EMSA was added to all reactions prior to complexes formation.

In all competition EMSA experiments, unlabeled competitor RNA (where present) was always first combined with 0.5–1 pmol of RNAP. Then, labeled RNAs were added and the mixtures (with an end-volume of 5 μl) were incubated at 37°C for 10–20 min and put on ice with 0.6 μl 1 mg/ml heparin (to dissolve non-specific complexes). When heparin was used as a negative control for complex-formation it was added to RNAP before this was mixed with RNA. After adding 2 μl 50% glycerol to each sample, complexes were separated on 5% PAA in 0.5× TBE at 200 V with some bromophenol blue loaded alongside as a migration marker. Gels were fixed, dried and analyzed as described above.

#### Transcription assays

*In vitro* transcription assays with purified RNAP were done essentially as described previously (29). In an end-volume of 8.5 μl, RNAP (1–2 pmol) was mixed with competitor (2–8 pmol RNA or heparin to 100 μg/ml), 1 μl 10× TB (200 mM Tris–HCl pH 7.8; 400 mM KCl; 10 mM MgCl_2_) and incubated for 5–10 min at room temp to let complexes form. Reactions were started by adding 1.5 μl of a mix containing 1 mM each of CTP, ATP, GTP, 0.1 mM UTP, 0.5 μl [α-^32^P]-UTP (3000 Ci/mmol; Hartmann), 1 mM CpA and template DNA with the T7A1 promoter and T7-tr2 terminator; yielding ∼100 nt-long and ∼170 nt-long products by termination and run-off transcription, respectively. After 5 min incubation at 37°C reactions were stopped with 10 μl formamide-containing loading buffer. Where indicated, DNA template was allowed to incubate with RNAP before inhibitor was added with the reaction mix. Tri-nucleotide formation as a test for open complex formation was done as above but the reaction mix only contained template DNA, 0.5 μl [α-^32^P]-UTP (3000 Ci/mmol; Hartmann), 0.2 μl 1 mM UTP and 0.05 μl 5 mM CpA. For the analysis of transcription elongation, elongation complexes containing 11 nucleotide-long RNA were obtained in the reaction as above except nucleotide mixture added to reaction contained 0.25 mM CpApUpC, 0.1 mM ATP, GTP and 0.5 μl [α-^32^P]-GTP (3000 Ci/mmol; Hartmann). Elongation complexes were allowed to form for 10 min followed by addition of 2–8 pmol competitor and all four NTPs to 200 μM final concentration, for 5–10 min at 37°C (28). Reactions were stopped with 10μl loading buffer and analyzed by denaturing PAGE and phosphorimaging.

### Purification of RNPs through IsrA

**Figure 1.**
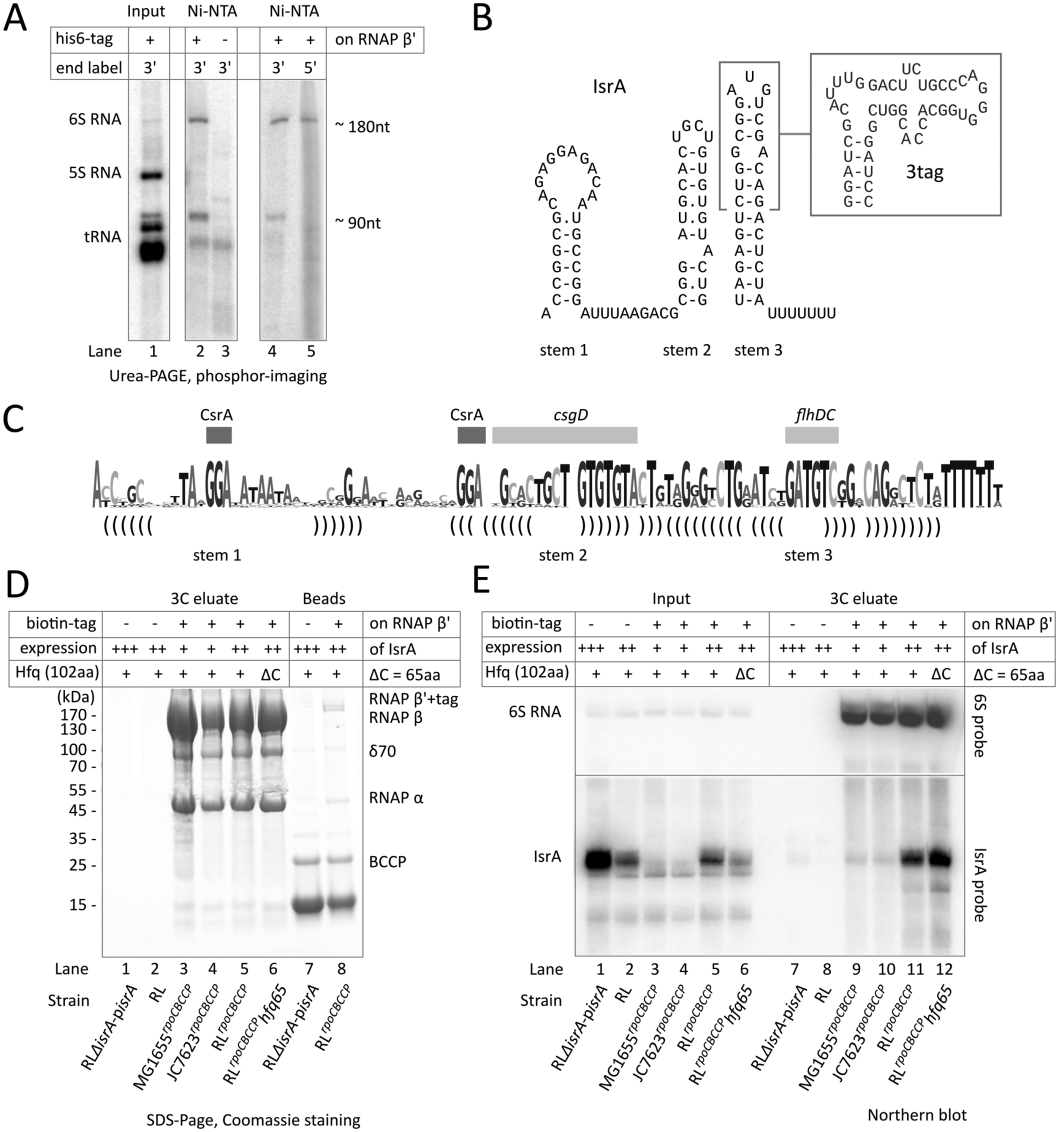
A small non-coding RNA, IsrA, co-purifies with RNA polymerase. (**A**) Pull-down of RNAs with 6xHis-tagged (strain RL*^rpoC^*^HIS^) or tag-less RNAP on Ni^2+^-NTA sepharose. Isolated RNAs were 5′- or 3′ end labeled and analyzed as described in the Materials and Methods. (**B**) Secondary structure model of the purified RNA, identified as IsrA ((23); a.k.a. IS61 (20) or McaS (21,22)) which was based on phylogenetic analysis (see Supplementary Figure S2). An extension that substitutes the outer portion of stem 3 and provides affinity to streptomycin (streptotag (32); see Figure 2) is boxed. (**C**) Summary of IsrA structure in sequence logo representation; height of nucleotides mark the degree of conservation. Base pairs that form stems are indicated by brackets; rectangles indicate regions implicated in protein binding (CsrA), or translational regulation of *flhDC*, or *csgD* mRNAs (21,22,43). (**D**) Purification of RNAP on streptavidin sepharose through a biotinylated tag at the β’ subunit that can be cleaved off using HRV3C (3C) protease. RNAPs were isolated from strains; lane 1: RLΔ*isrA*-p*isrA* (tag-less RNAP, IsrA under mutant promoter on multi-copy plasmid); lane 2: RL (tag-less RNAP, genomic IsrA under mutant promoter); lane 3: MG1655*^rpoC^*^BCCP^ (BCCP-tagged RNAP, genomic IsrA under wild-type promoter); lane 4: JC7623*^rpoC^*^BCCP^ (BCCP-tagged RNAP, genomic IsrA under wild-type promoter); lane 5: RL*^rpoC^*^BCCP^ (BCCP-tagged RNAP, genomic IsrA under mutant promoter); lane 6: RL*^rpoC^*^BCCP^*hfq65* (BCCP-tagged RNAP, genomic IsrA under mutant promoter, Hfq lacks C-terminus) (see also Supplementary Table). Relative expression levels of IsrA are shown above the gels (expressed from wild-type promoter in chromosomal locus (+), expressed from a mutant promoter in the chromosomal locus (++) or from mutant promoter from a high-copy plasmid (+++)). RNAPs were released by HRV3C (3C) cleavage and analyzed by SDS/PAGE on a 4–20% gradient gel. Strains that have RNAP without a biotinylated tag were used as a control. Lanes 7 and 8 are controls for non-specific binding to and efficiency of release from beads of RNAP in lanes 1 and 5, respectively. (**E**) Northern blot analysis of RNAs that co-purified with biotinylated RNAP in panel (D). The blots were probed against IsrA (bottom) or 6S RNA (top). Indicated are the presence of the biotinylated affinity tag on RNAP β’ (+ or – tag), the expression levels of IsrA in the input extract (as in panel (D)), and whether Hfq in the cells was truncated to its core of 65 amino acids (ΔC) or was wild type (+).

Cell extracts from strains RL or RL*^rpoC^*^CBBP^Δ*isrA-*p*isrA^3tag^* (Supplementary Table), the latter transformed with pGemT-*IsrA^3tag^* expressing IsrA with an optimized ‘StreptoTag’ (32) built into its 3′-terminal stem (Figure 1B, Supplementary Figure S1F), were prepared as described above in BB @ 200 mM KCl. RNPs were purified using dihydrostreptomycin (Sigma) coupled to epoxy-activated sepharose 6B (Sigma) following the procedures of (33,34), and, after extensive washing with BB @ 250 mM KCl, eluted with 200 μM streptomycin (Melford) in BB @ 200 mM KCl. RNAs were isolated from the eluate by phenol-chloroform extraction and ethanol precipitation; proteins by TCA-precipitation. Proteins co-purified with IsrA-3tag RNA were visualized by 4–20% SDS-PAGE. For LC/MS, triplicate samples were run into the gel for 1–1.5 cm, and cut-out in one slice each. Proteins were reduced with DTT (Sigma), alkylated with iodoacetamide (Sigma) and digested with modified trypsin (Promega). The digests were analysed by LCMSMS using a Dionex U3000 nano-HPLC system (Thermo) coupled to an Orbitrap LTQ XL(ETD) (Thermo) mass spectrometer. Peptides were separated on a 25 cm x 75 μm PepMap column (Thermo) using a 67 min water acetonitrile gradient (0.08% formic acid). Precursor ions were detected in positive mode at 350–1600 *m*/*z* with a resolution of 60 000 (at 400 *m*/*z*) and a fill target of 500 000 ions and a lockmass was set to 445.120023 *m*/*z*. The 10 most intense ions of each MS scan (with a target value of 10 000 ions) were isolated, fragmented and measured in the linear ion trap. Peaklists in the Mascot generic format (*.mgf) were generated using msconvert (proteowizard.sourceforge.net) and the *E. coli* K-12, substr. MG1655 genome was searched using X!Tandem and the gpm interface (thegpm.org; version 2013.09.01.1) with carbamidomethyl set as a fixed modification and Met oxidation set as a variable modification. Two refinement steps were included in the search to include deamidation and methylation artefacts. The protein level false positive rate (see: http://wiki.thegpm.org/wiki/False_positive_rate) for all three repeats was <1%. The raw data have been deposited at www.proteomeXchange.org.

### Miscellaneous

#### Protein analysis

Proteins, taken up in 1 vol 2× loading buffer (2% SDS, 80 mM Tris–HCl pH 6.8, 10% glycerol, 10 mM EDTA, 0.1% Bromophenol Blue, 200 mM DTT) were analyzed on RunBlue precast 4–20% SDS-PAGE (Expedeon) and stained with InstantBlue (Expedeon), a Coomassie-based solution; Silver staining was done with SilverXpress (LifeTechnologies). RNAP, partially purified over a heparin column from BW-62, was used as a marker for RpoA, B and C subunits.

#### Congo-red assay

For visualization of the amount of curli present in the cell walls of stationary cells, 10-fold serial dilutions of cells were spotted on LB agar without NaCl containing 20 μg/ml congo red and 10 μg/ml brilliant blue (22) and grown at room temperature for 48 h. Plates were scanned through the bottom against a dark background on an Epson Perfection V700 Photo with ‘Color restoration’ set. For contrast enhancement, levels were uniformly changed and the non-relevant background converted to black with the GNU Image Manipulation Program (www.gimp.org).

#### Phylogenetic analysis

Sequences for sRNAs or ORFs, including 200–300 bp up- or downstream, were retrieved from EcoCyc (http://ecocyc.org) and homologs identified by iterative Blast searches (with filtering off and expectation >10) against (selected) bacterial genomes at https://asap.ahabs.wisc.eduasap/sim_search_query.php or http://www.ncbi.nlm.nih.gov/sutils/genom_table.cgi?organism=microb (when a choice of organisms from a list was still available). Alignments were prepared with CLC Sequence Viewer 6.9 (http://www.clcbio.com) using ClustalW (http://www.clustal.org/clustal2/) or MUSCLE (http://www.drive5.com/muscle/) algorithms, MAFFT (http://mafft.cbrc.jp/alignment/server) and Jalview 2.8 (http://www.jalview.org/); secondary structures were analysed with RNAfold or RNAalifold (http://rna.tbi.univie.ac.at/) and promoter sequence specificity for the IsrA gene was assessed with RegulonDB (http://www.ccg.unam.mx/Computational_Genomics/PromoterTools/). Sequence logos were created at http://weblogo.berkeley.edu.

## RESULTS

### A small non-coding RNA IsrA co-purifies with RNA polymerase

Recent examples of non-coding RNAs that interfere with transcription by RNA polymerase (RNAP) (17) stimulated us to check whether in bacteria other RNAs than 6S RNA can regulate the activity of this multi-subunit enzyme. In a preliminary experiment we bound cellular RNAP to a Ni^2+^-NTA sepharose column by means of a 6× histidine affinity-tag attached to the C-terminus of the β’ subunit (chromosome encoded; strain RL*^rpoC^*^HIS^, see Supplementary Table). After multiple washing steps (using solutions containing 200 mM KCl, 10 mM imidazole and 100 μg/ml heparin) RNAs associated with the bound fraction were purified. Two RNAs, of about 180 and 90 nt, were isolated which were not present in preparations from cells expressing untagged RNAP β’ (Figure 1A, cf. lanes 2 and 3). After attaching a linker to their 3′ ends (see Materials and Methods), the RNAs were amplified by RT-PCR, cloned and sequenced. This identified the two RNAs as 6S RNA (15,31,35) and IsrA (Figure 1B, C)(23) which was confirmed by Northern blotting (e.g. Figure 1E). RNA degradation or processing in the extracts after breaking the cells, was limited: cDNA products were obtained that covered the complete IsrA sequence (94 nt, including the 7 U's at the 3′ end) and partially processed forms of 6S RNA (of 191–197 nt) which, in its mature form, is 183 nt. Furthermore, both 6S and IsrA could be labeled at their 3′ ends (Figure 1A, lane 4), indicating the absence of a 3′ phosphate characteristic of hydrolytic degradation. The 5′ end of IsrA is protected from phosphorylation by T4 PNK likely by a triphosphate group characteristic for primary transcripts, whereas processed 6S RNA was phosphorylatable (Figure 1A, lane 5). Both 6S RNA and IsrA could be released from Ni^2+^-NTA sepharose by proteinase K digestion, indicating that a protein (likely RNAP) determines the binding of these RNAs to the matrix.

The majority of cloned cDNAs derived from IsrA and 6S RNA, which indicated specific interactions. Protein analysis of the fraction that remained bound to the nickel column, however, showed that a number of other proteins were isolated together with RNAP. To ensure that IsrA bound specifically to RNAP, the purification was repeated using an alternative affinity-tag on RNAP β’. For this, we used the biologically active C-terminal region of the biotin carboxyl carrier protein (BCCP; (24)) which was linked to RNAP β’ via a peptide containing the cleavage site for the human rhinovirus 3C (HRV3C) protease (Supplementary Figure S1A; strain RL^*rpoC*BCCP^). Extracts were prepared from cells grown in the presence of biotin and mixed with streptavidin sepharose beads. After extensive washes in the presence of 100 μg/ml heparin, RNAP that was bound to the beads was released by HRV3C cleavage. This procedure yielded large amounts of RNAP from strain RL^*rpoC*BCCP^ (Figure 1D, lane 5). Northern blot analysis confirmed that both 6S RNA and IsrA co-purified with RNAP (Figure 1E, lane 11). In absence of tagged RNAP no significant binding of proteins or RNAs to streptavidin sepharose was observed as shown by control purifications using the parental strain (RL), or a derived strain in which IsrA was overexpressed from a high-copy plasmid (RLΔ*isrA*-p*isrA*) (Figure 1D, lane 1, 2 and Figure 1E, lanes 7, 8). We therefore conclude that IsrA specifically co-purifies with RNAP.

We noted differences in growth-dependent expression of IsrA in the RL-based strains used above (RL*^rpoC^*^HIS^, RL*^rpoC^*^BCCP^) and the commonly used MG1655, which was explained by a point mutation in the IsrA promoter (see Supplementary Text). However, we showed that IsrA readily co-purified with BCCP-tagged RNAP from MG1655*^rpoC^*^BCCP^ and JC7623*^rpoC^*^BCCP^ (the mutation-less parent of RL strains) cells (Figure 1D, E; Supplementary Text and Supplementary Table), demonstrating that the association of IsrA with RNAP is strain-independent.

Bioinformatics analysis showed that IsrA appears to be expressed in a subset of enterobacteria (Supplementary Figure S2) and can be modeled to adopt a secondary structure with three stem-loops (Figure 1B). The 5′ third of the molecule covering stem 1 is highly variable and only alignments that included the promoter region revealed the putative 5′ ends of IsrA homologs (Supplementary Figure S2A). Phylogenetic analysis, backed up by lowest-energy calculations (Supplementary Figure S2C) suggested two stems in the 3′ portion of the sRNA which stand out by conservation in the 3′ half of stem 2 and the loop of stem 3 (Figure 1C; Supplementary Figure S2B). As will be seen below, stem 2 determines specific interaction of IsrA with RNAP.

### *In vivo* IsrA exists as an RNP with proteins S1, Hfq, ProQ, PNPase, CsrA and RNAP

To further investigate association of IsrA with RNAP we used a reciprocal approach, by pulling down proteins from the cell via a tagged IsrA. This approach would also provide information on the proteins associated with an IsrA/RNAP complex. Because we found that stem 3 was not essential for the interaction with RNAP (see below) this stem-loop was partially substituted with an affinity tag, streptotag, that specifically binds to streptomycin (32) (Figure 1B). Tagged IsrA was expressed from its own promoter on a multi-copy vector in a strain lacking the endogenous IsrA (RL*^rpoC^*^BCCP^Δ*isrA*-p*isrA^3tag^*, Supplementary Table). Full-length IsrA-3tag RNA co-purified with biotinylated RNAP on streptavidin sepharose (Supplementary Figure S3), indicating that the tag neither inhibited the interaction with RNAP nor seriously affected termination of IsrA transcription.

**Figure 2.**
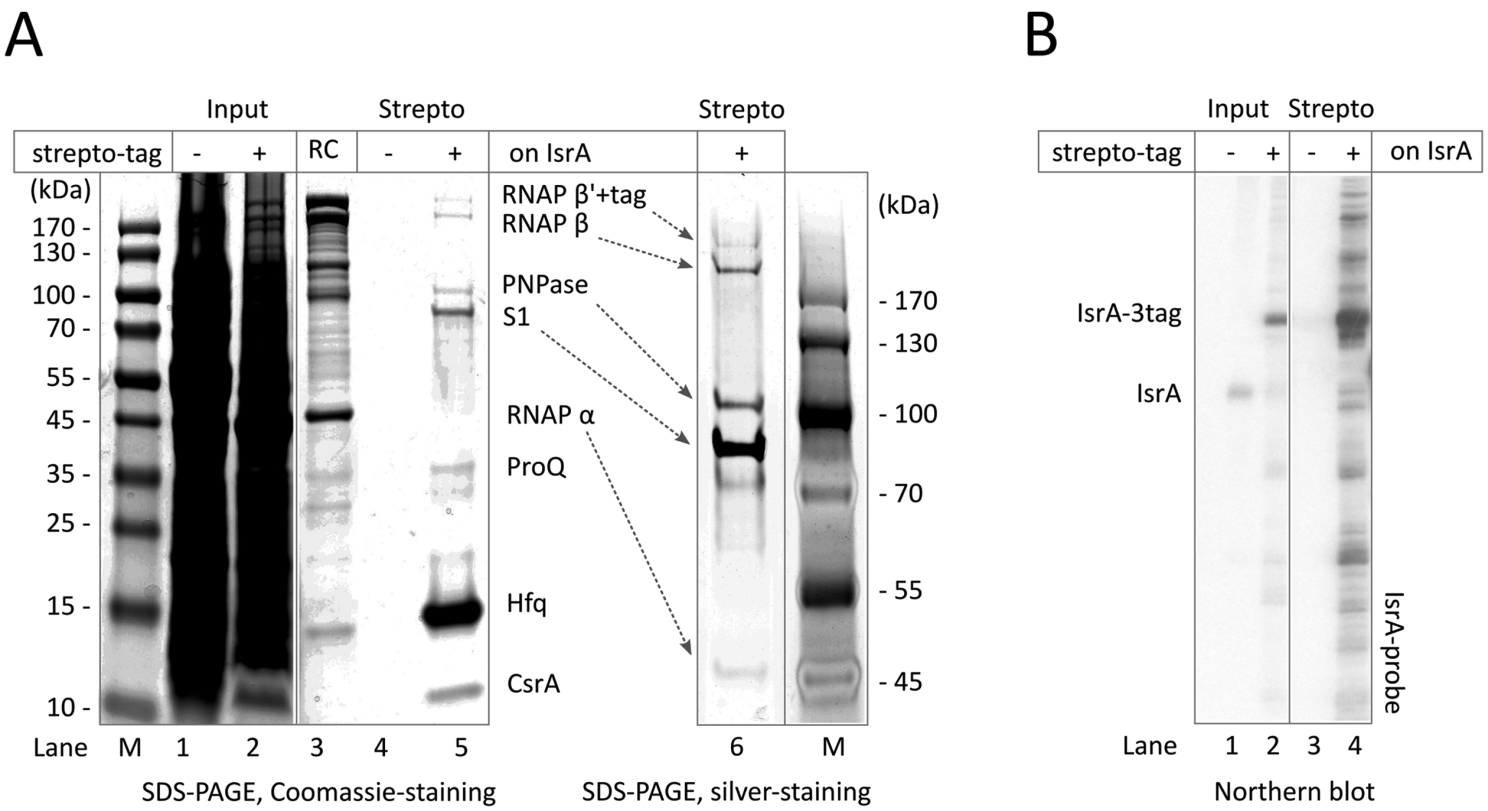
RNAP and a defined set of proteins co-purify with IsrA carrying a streptotag. (**A**) Streptotagged IsrA-3tag (see Figure 1B) and proteins associated with it were isolated on di-streptomycin beads from strain in which the endogenous gene for IsrA was disrupted (strain RL*^rpoC^*^BCCP^ Δ*isrA*-p*isrA3tag*, see Supplementary table). Co-purifying proteins (Strepto lanes) were analyzed by SDS-PAGE on 4–20% gradient gels and stained with Coomassie or silver as described in the Materials and Methods. Mock isolation with the di-streptomycin beads using the parental strain with untagged IsrA (RL; lane 4) was used as a negative control. The presence and absence of affinity tags on IsrA is indicated by + and –, respectively. RNAP purified on a heparin column from strain BW-62 was used as size control (RC; lane 3). (**B**) RNA was analyzed by Northern blotting with an IsrA-specific probe. Note that the blot is overexposed to make the IsrA band in the input visible. The presence and absence of affinity tags on IsrA is indicated by + and –, respectively.

From the same cells we purified tagged IsrA on a di-streptomycin-matrix (13,34) (Figure 2B). As described previously (13), non-specific binding of proteins to the di-streptomycin-matrix was negligible (Figure 2A, lane 4). Interestingly, only a limited set of proteins co-purified with streptotagged IsrA (Figure 2A, lanes 5, 6). Using GeLCMSMS, we analyzed all the proteins that co-purified with IsrA. This resulted in high confidence identifications of several proteins in the major bands that are visible on the SDS-PAGE gel (Figure 2A; and Table 1, and Supplementary Data SD1). Indeed, subunits of RNAP (α, β and β’) were present in the pull-down in abundance (even clearly visible in the SDS gels; Figure 2A, lanes 5 and 6), supporting our conclusions that IsrA specifically associates with RNAP. Intriguingly, however, we observed other proteins in amounts comparable to RNAP subunits (Table 1), represented by RNA chaperones Hfq (36) and ProQ (37,38), protein S1 (39), PNPase (40,41), and CsrA (42). CsrA and Hfq, but no other proteins, were previously shown to associate with IsrA (21,43). Less represented proteins that were detected by mass-spectrometry comprised some ribosomal proteins (r-proteins), tRNA modification enzyme DusA (44), poly(A) polymerase (PAP I; (45,46)), RNase G (47), a subunit of the membrane associated ATP-synthase, the Lon protease, lipoprotein Entericidin B, and putative transcription factor YhgF. Overall, the limited number of proteins that co-purified with IsrA indicate that this RNA forms particular complexes, at least one of which involves RNAP. Interestingly, no σ-subunit of RNAP was found in the pull-down, suggesting that IsrA may regulate core enzyme of RNAP, an idea signified by our finding that IsrA can specifically interact with core (see below).

**Table 1.** Proteins co-purifying with streptotagged IsrA-3tag were assigned based on comparison and identification by mass-spectrometry, done in triplicate

Accession	Ecoli #	Gene	Protein	Mr	log(e)	#	Description
gi|16128878	b0911	rpsA	S1	61.1	−299.83	125	30S r-protein S1; translation initiation factor.
gi|16131994	b4172	hfq	Hfq	11.2	−296.6	362	RNA chaperone; host factor for phage Q β replication.
gi|49176156	b1831	proQ	ProQ	25.9	−238.23	128	RNA chaperone; regulator of ProP expression.
gi|145698316	b3164	pnp	PNPase	77.1	−130.1	44	polynucleotide phosphorylase or - polymerase.
gi|16131817	b3987	rpoB	RNAP β	150.5	−106.33	33	RNA polymerase; β subunit.
gi|16131174	b3295	rpoA	RNAp α	36.5	−66.87	28	RNA polymerase; α subunit.
gi|16131602	b3734	atpA	AtpA	55.2	−66.33	23	F1 sector of membrane-bound ATP synthase.
gi|90111550	b3162	deaD	CsdA	70.5	−65.8	22	ATP-dependent RNA helicase.
gi|16130603	b2696	csrA	CsrA	6.9	−57.03	38	Regulatory protein for carbon source metabolism.
gi|16131818	b3988	rpoC	RNAP β'	155.1	−51.13	19	RNA polymerase; β' subunit.
gi|145698340	b4049	dusA	DusA	36.8	−42.73	16	tRNA-dihydrouridine synthase A.
gi|16128424	b0439	lon	Lon	87.4	−41.2	17	DNA-binding ATP-dependent protease.
gi|90111563	b3247	rng	RNaseG	55.3	−12.93	7	(Endo) ribonuclease G.
gi|162135892	b0143	pcnB	PAP I	53.8	−12.4	9	Poly(A) polymerase.
gi|49176463	b4411	encB	EncB	4.8	−12.4	3	Entericidin B; translationally repressed by MicA.
gi|90111588	b3407	yhgF	YhgF	85.1	−11.9	4	Has C-terminal S1; predicted to assist transcription.
gi|16131143	b3255	accB	BCCP	16.7	−9.23	4	C-term of BCCP, used as an affinity tag on RNAP β'.
gi|16131220	b3341	rpsG	S7	20	−73.23	30	30S r-protein S7.
gi|16131814	b3984	rplA	L1	24.7	−62.23	26	50S r-protein L1, also translational regulator.
gi|16131193	b3314	rpsC	S3	26	−50.23	22	30S r-protein S3.
gi|16131182	b3303	rpsE	S5	17.6	−40.9	17	30S r-protein S5.
gi|16131177	b3298	rpsM	S13	13.1	−40.8	20	30S r-protein S13.
gi|16131120	b3230	rpsI	S9	14.8	−40.17	19	30S r-protein S9.
gi|16131175	b3296	rpsD	S4	23.5	−39.4	24	30S r-protein S4, also translational regulator.
gi|16132025	b4203	rplI	L9	15.8	−36.17	13	50S r-protein L9.
gi|16131180	b3301	rplO	L15	15	−35.57	13	50S r-protein L15.
gi|16128162	b0169	rpsB	S2	26.7	−25	15	30S r-protein S2.

Their molecular mass in kDa (Mr), the average base-10 log of the expectation that any particular protein assignment was made at random (log(e)) and the total number of peptides (#) identified for each protein are indicated.

### Interaction of IsrA with RNAP does not depend on Hfq-mediated mRNA(s) binding *in vivo*

Hfq, a general RNA chaperone that confers stability to sRNAs that terminate in a rho-independent manner (36,48,49), was overrepresented in the above pull-down through tagged IsrA. As reported previously (22), in strains lacking Hfq, IsrA could not be detected indicating that Hfq is required for IsrA stability (not shown). Note also that the majority of IsrA isolated via the streptotag remained intact with only minor nicked species (Figure 2B), suggesting that most of IsrA in the cell exists as an RNP, in which it is associated with at least Hfq. The N-terminal 65 amino acids of Hfq are sufficient to support formation of the hexameric Hfq-ring and the binding to sRNAs, while the remaining C-terminal tail is required for the binding of two RNAs and possibly their annealing (50–52). It is possible that IsrA is associated with RNAP via Hfq-mediated interaction with mRNAs. However, deletion of the C-terminal tail from Hfq (strain RL*^rpoC^*^BCCP^*hfq65*) did not affect the stability of IsrA or its association with RNAP (Figure 1E, lanes 6 and 12). IsrA is a major player in the control of curli-synthesis (21,22,53), because when there is no IsrA present, curli (red-color in Supplementary Figure S4) is produced. The C-terminus of Hfq is also essential for IsrA-controlled regulation of translation; curli-synthesis was not inhibited in cells having the truncated form of Hfq (Supplementary Figure S4). These results indicate that the C-terminal domain of Hfq, while being required for IsrA function in the cell, possibly via assisting base pairing with target mRNA, is not needed for the association of IsrA with RNAP. The result cannot exclude association of IsrA with RNAP through its interaction with mRNA that potentially may also be pulled down with RNAP. However, as will be seen below IsrA can specifically associate with purified core enzyme of RNAP.

### IsrA binds specifically to core and holo RNAP *in vitro*

**Figure 3.**
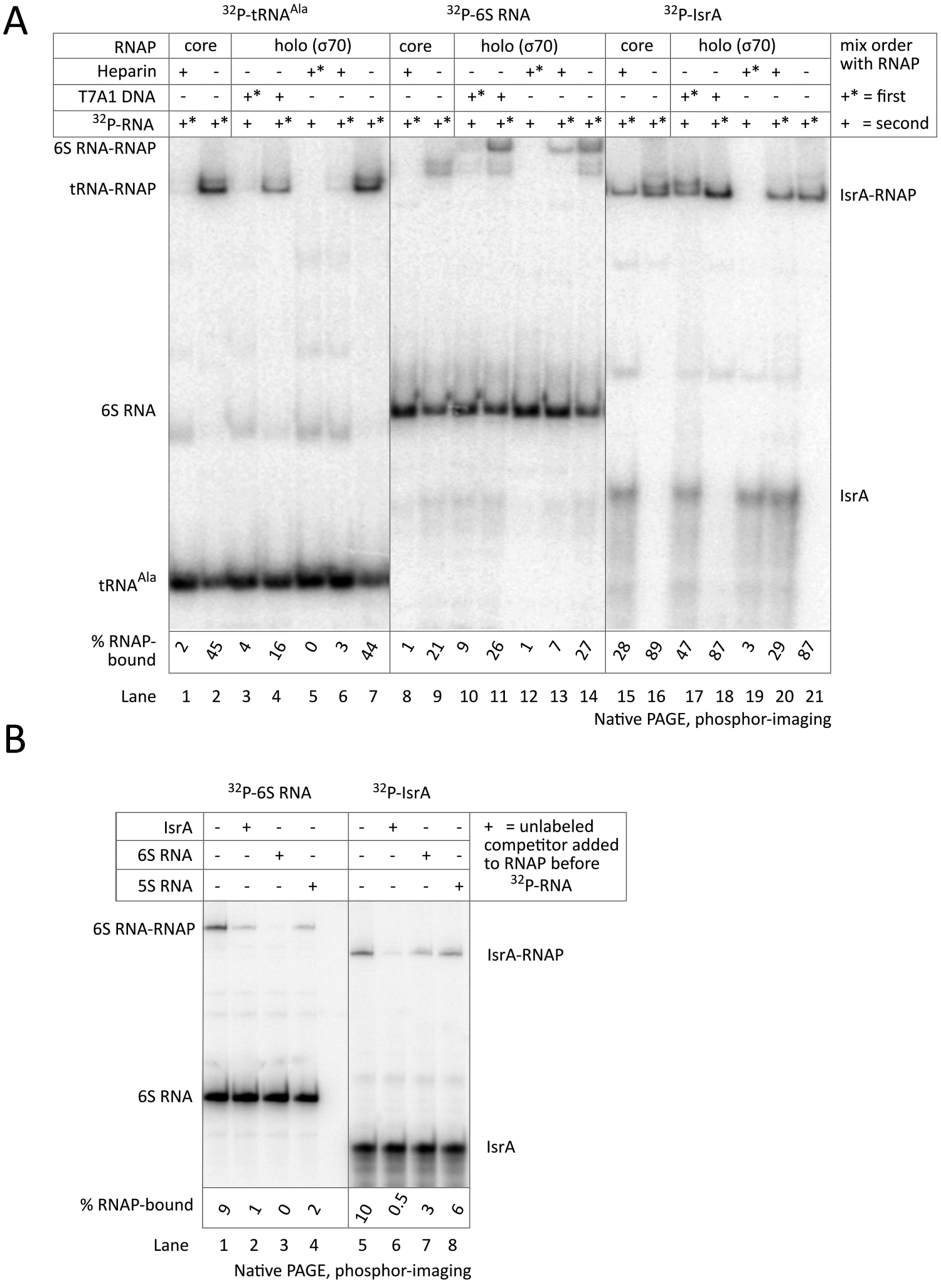
IsrA specifically binds core enzyme of RNAP. (**A**) Electrophoretic mobity shift assay (EMSA) with radioactively labeled, gel-purified T7 synthesized ^32^P-labelled RNAs (tRNA^Ala^, 6S RNA or IsrA) which were mixed with equimolar amounts of purified core or holo (σ^70^) enzymes of RNAP. The labeled RNA and a competitor (T7A1 promoter DNA or heparin) were added to RNAP first (+*) or second (+). (**B**) Competition of various unlabeled RNAs with ^32^P-labeled 6S RNA and IsrA. 15-fold excess of unlabeled competitor RNA (Supplementary Figure S5) was added to RNAP before addition of labeled RNAs. All reactions contained total yeast RNA as a non-specific competitor prior to complex formation. Heparin was added to all reactions before loading on the gel to dissolve non-specific complexes.

During the pull-down experiments described above, the interaction of IsrA with RNAP can be indirect and be mediated by other proteins that co-purify with IsrA, such as Hfq that has been isolated with RNAP (12). In order to investigate this possibility we synthesized IsrA *in vitro* by means of T7 RNAP and, after labeling and gel-purification, analyzed its binding to purified core and holo RNAPs by electromobility-shift assays. Core enzyme and σ^70^ were purified to apparent homogeneity using stringent procedures (28), and mass-spectrometry did not detect any contaminating proteins such as Hfq. As negative and positive controls, binding reactions of RNAP with tRNA^Ala^ and 6S RNA were set up (Figure 3).

Both, RNAP core and holo enzymes bound either of the RNAs (Figure 3A, lanes 2, 7 and 9, 14 and 16, 21). In fact, IsrA was bound more efficiently than 6S or tRNA^Ala^ (which we attribute to two modes of binding, specific and non-specific; see below). However, RNAP has a high affinity for any nucleic acid, and to increase stringency for complex formation we used the polyanion heparin. Heparin tightly binds to RNAP and causes non-specific and/or weak complexes to fall apart, even if added after complex formation. As expected, when heparin is added before the RNA, no complex formation is observed even with holo RNAP (Figure 3A, lanes 5, 12 and 19). tRNA^Ala^ that does not bind RNAP specifically lost its association with either core or holo RNAPs on subsequent exposure to 100 μg/ml heparin (Figure 3A, compare lanes 1 and 6 to 2 and 7). In agreement with published data (31,35), we found that 6S RNA only forms specific, heparin-resistant complexes with the holo enzyme (Figure 3A, compare lanes 8 and 13 to 9 and 14). Surprisingly, IsrA formed heparin-resistant, i.e. specific, complexes with both core and holo enzymes (Figure 3A, compare lanes 15 and 20 to 16 and 21).

6S RNA mimics nucleic acids in the promoter open complex, while tRNA^Ala^ likely binds in the main channel of RNAP. Accordingly addition of a strong T7A1 promoter to RNAP prior to tRNA^Ala^ or 6S prevented or diminished formation of complexes with these RNAs (Figure 3A, compare lanes 3 and 10 to 7 and 14). In contrast, while addition of T7A1 before IsrA destructed part of the complexes as did heparin, a large amount of IsrA was still able to form a complex with RNAP (Figure 3A, compare lane 17 to 21). This result suggested that the specific IsrA binding site on RNAP is different from that of promoter DNA and 6S RNA. This was confirmed by competition experiments in which an excess of unlabeled 6S RNA prevented association of labeled 6S RNA with RNAP (Figure 3B, lane 3) but did not interfere with the binding of labeled IsrA (Figure 3B, lane 7). Reciprocally, unlabeled IsrA could replace only labeled IsrA (Figure 3B, lane 6). Note that, in all reactions contained total yeast RNA as a non-specific competitor, and heparin was added to all reactions after complex formation, thus abolishing non-specific complexes of RNAs with RNAP. Accordingly, an extra presence of non-specific competitor 5S rRNA made no difference to RNAP complex formation of either 6S RNA or IsrA (Figure 3B, lanes 4, 8). We conclude that IsrA can directly interact with RNAP at a site not required for or interfering with promoter or 6S RNA binding, i.e. away from the main nucleic-acids-binding channel of RNAP.

We also noted that *specific* 6S RNA/holo complexes migrated considerably slower than non-specific 6S RNA/core or specific IsrA/core and IsrA/holo complexes, which between them had similar mobility in the gel. It is possible that, because of the different binding sites of 6S RNA and IsrA, specific 6S RNA/holo complexes have a different conformation to those of IsrA/holo and IsrA/core, which changes the migration in the gel. Another possible explanation is that specific 6S RNA/holo complexes migrate more slowly due to the presence of the σ^70^ subunit, while IsrA may induce the release of the σ^70^ subunit from the holo RNAP (and turn the complex into IsrA/core). However, both possibilities require further investigation.

### Stem 2 of IsrA is needed for specific interaction with RNAP

**Figure 4.**
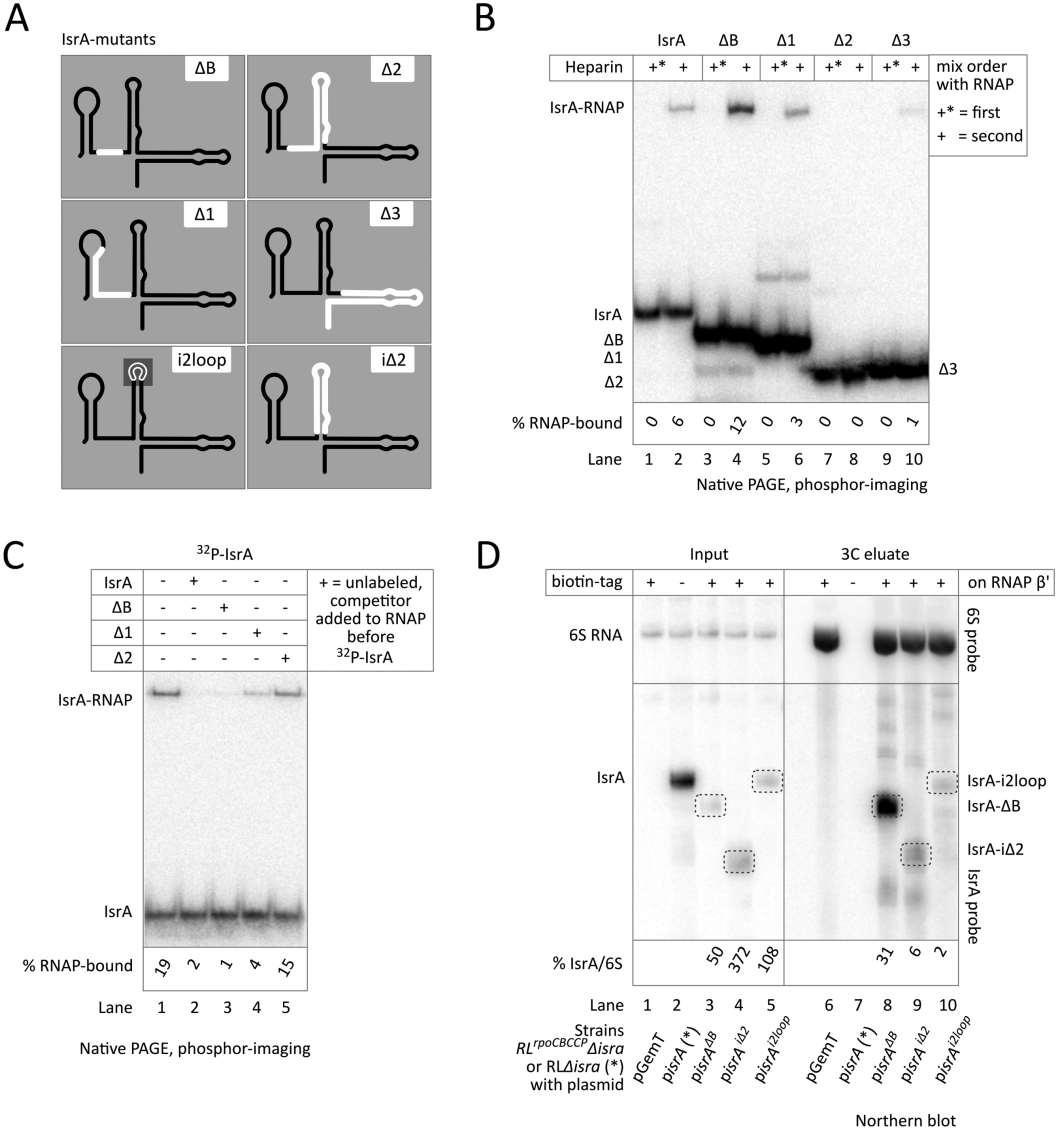
Stem 2 of IsrA is required for specific binding to RNAP. (**A**) Mutant versions of IsrA (see also Supplementary Figure S1F) shown as a black line-diagram with deleted regions indicated in white. In the i2loop mutant the loop closing stem 2 has been replaced with GAAG (gray; see also Supplementary Figure S6A). (**B**) EMSA of RNAP and mutant labeled IsrA RNAs with heparin added before (+*) or after (+) complex formation. All reactions contained total yeast RNA as a non-specific competitor prior to complex formation. (**C**) Competition of unlabeled mutant IsrA RNAs (15-fold molar excess) with labeled wild-type variant. Unlabeled competitor RNA was always added before complex formation of RNAP with labeled RNA. All reactions contained total yeast RNA as a non-specific competitor prior to complex formation. Heparin was added in all reactions after formation of complexes. Only IsrA mutants that are able to bind RNAP in the presence of heparin (see panel B) compete against association of labeled IsrA. (**D**) Northern blot analysis of mutant IsrA RNAs that co-purified with RNAP (for protein gel see Supplementary Figure S6B. Plasmids from which the mutant IsrA RNAs (see panel A) were expressed, along with an empty vector (pGemT), were transformed into strain RL*^rpoC^*^BCCP^Δ*isrA* with a disrupted IsrA gene and carrying biotinylated RNAP. A strain expressing RNAP without the biotinylated tag (RLΔ*isrA-*p*isrA*(*)) was used as a negative control (input and pull-down are overloaded). sRNAs were released from beads with 3C, and the blots were probed against IsrA (bottom) or 6S RNA (top). Below the blot, quantitation of the bands corresponding to IsrA mutants (encircled in the blot), normalized to the corresponding bands of 6S RNA.

The finding that IsrA could only compete against itself for RNAP association suggested that particular structural elements of this sRNA are responsible for this interaction. By testing a set of IsrA mutants (Figure 4A; Supplementary Figures S1F, S6A, S5) we obtained evidence that stem 2 is important for the *in vitro* binding of IsrA to RNAP. Whereas disruption of the linker between stems 1 and 2 (ΔB) or of stem1 (Δ1) had no negative effect on complex formation with RNAP, this was completely abolished on removal of stem 2, while a comparably large deletion of stem 3 (Δ3) had much less effect (Figure 4B). Note that only specific interactions of IsrA with RNAP were monitored in this experiment, as heparin was present in all reactions. Competition experiments showed that only mutants that bound RNAP were able to compete for the IsrA binding site with full-length labeled IsrA, and to an extent that correlated with their RNAP affinity (Figure 4C); the linker mutant (ΔB) could fully compete, the stem 1 disruptant (Δ1) partially and the sRNA lacking stem 2 (Δ2) not at all.

To test whether stem 2 of IsrA determines interaction with RNAP *in vivo*, we either removed stem 2 or introduced mutations in loop 2 of the overexpressed IsrA (strains RL*^rpoC^*^BCCP^Δ*isrA*-p*isrA^iΔ2^*, RL*^rpoC^*^BCCP^Δ*isrA*-p*isrA^i2loop^*), and compared their co-purification with RNAP to mutant IsrA lacking the single stranded region separating stems 1 and 2 (strain RL*^rpoC^*^BCCP^Δ*isrA*-p*isrA^ΔB^*), which does not affect RNAP binding *in vitro*. As seen from Figure 4D while the linker deletion was neutral, mutations in or removal of stem 2 prevented enrichment of the mutant IsrA in the RNAP fraction. This result corroborates our *in vitro* data that IsrA specifically interacts with RNAP via its stem 2.

### Only non-specific interaction of IsrA with RNAP inhibits initiation of transcription *in vitro*

**Figure 5.**
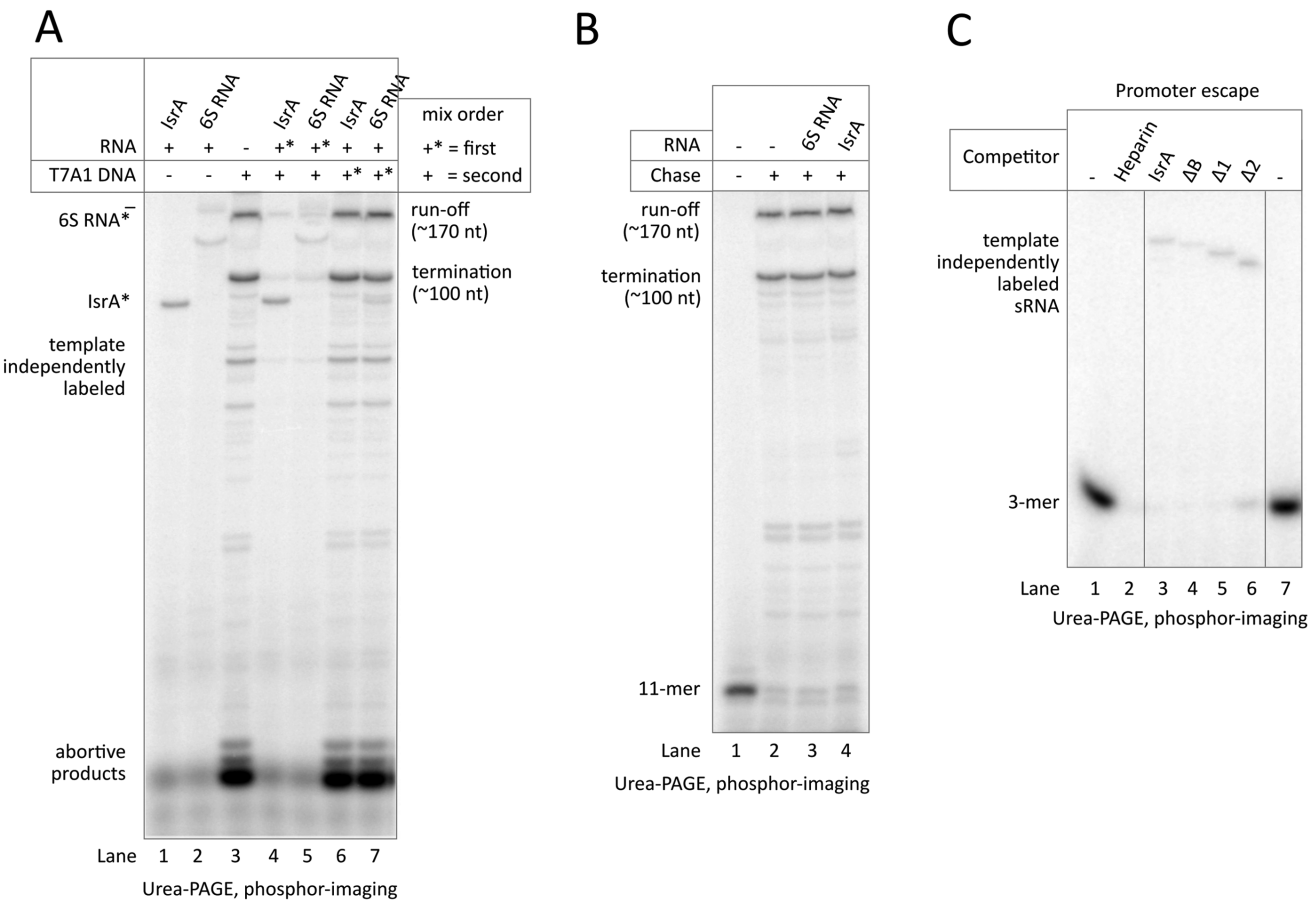
Only non-specific binding of IsrA to RNAP can inhibit transcription. (**A**) Transcription on a linear template containing the T7A1 promoter. The order in which RNAP was allowed to associate with the promoter DNA or competitor RNA is given (+* added first; + second). Note the template-independent labeling of IsrA at the 3′ end by RNAP, which suggests non-specific binding near the active center of RNAP (see text). (**B**) Pre-formed 11-mer elongation complexes were chased in the presence or absence of sRNAs. (**C**) Abortive synthesis of CpApU on T7A1 promoter. Mutant IsrA RNAs or heparin were added before promoter DNA. Note that the IsrA variant Δ2, which cannot bind RNAP specifically, still inhibits initiation and is labelled at the 3′ end (see text for details).

We observed considerable non-specific binding of IsrA to RNAP (Figure 3A, lanes 16, 18 and 21), which was readily abolished by pre-incubation of RNAP with promoter DNA or by treatment of IsrA-RNAP complexes with heparin (Figure 3A, lanes 15, 17 and 20). However, addition of promoter DNA after these non-specific complexes formed did not lead to their destruction (Figure 3A, cf. lanes 17, 18). Accordingly, we observed that addition of IsrA before the promoter in the *in vitro* transcription reaction resulted in inhibition of transcription (Figure 5A lane 4), similar to the expected inhibition observed after pre-incubation of RNAP with 6S RNA (Figure 5A, lane 5). The stringency of IsrA binding in *in vitro* transcription could not be tested by addition of heparin (as we did in all other experiments), because heparin would inhibit transcription on its own. However, the following observations argue that the heparin-resistant specific binding of IsrA to RNAP does not affect binding of promoter DNA: (i) Stem 2 of IsrA is required for specific, heparin-resistant binding to RNAP (Figure 4B and C). However, Δ2 IsrA is still able to inhibit transcription initiation when added before promoter, indicating that it is the non-specific mode of binding of IsrA that is responsible for inhibition (Figure 5C, lane 6); (ii) In the presence of radioactive NTPs, IsrA was radiolabelled at the 3′ end by RNAP in a template-independent manner, suggesting its binding at the active center cleft, which likely causes inhibition of initiation. However, such end-labeling was also observed with Δ2 IsrA, which cannot bind RNAP specifically (Figure 5C). In addition, if the 3′ end of IsrA was binding specifically in the active site of RNAP then one would expect the 3′ end of IsrA to be strictly required for binding. This however is not the case as the mutant IsrA lacking the whole 3′ proximal part (Δ3 in Figure 4A) is still able to form heparin-resistant complexes with RNAP (Figure 4B, lane Δ3). (iii) Unlike any other known sRNA binding to RNAP, IsrA is able to bind RNAP after open complex formation and, apparently, not to compete with 6S RNA (Figure 3A, lane 17 and Figure 3B, lanes 2, 7), indicating that its binding site is different from the main nucleic-acids-binding cleft of RNAP. Accordingly, no 3′ end labeling has been seen when IsrA was added after open complex formation (Figure 5A, lane 6). This mode of IsrA binding however did neither affect transcription initiation nor elongation (Figure 5A, lane 6; B, lane 4).

These facts indicate that the observed inhibition of transcription initiation is caused by non-specific binding of IsrA to RNAP, which apparently interferes with promoter DNA binding. In the cells, however, IsrA likely exists as an sRNP (see above), which would suppress non-specific binding to RNAP. In agreement with this idea, mutations in or deletion of the Stem 2 of IsrA (which determines only specific interaction) strongly diminished co-purification with RNAP from cells in comparison to mutant ΔB (Figure 4D, cf. lane 8 to 9, 10) which strongly bound RNAP in vitro (Figure 4B, lane 4). This indicates that predominant in cells is the specific, stem 2-mediated, interaction of IsrA with RNAP that does not interfere with initiation of transcription. This further is supported by the fact that overexpression of IsrA in cells does not affect their growth (e.g. Supplementary Figure S4, row 5).

## DISCUSSION

Here, we present evidence that a small non-coding RNA, IsrA, specifically associates with core enzyme of RNAP (via its stem 2) and does so at a different site and mode than 6S RNA. This is the first example of specific interaction by a sRNA and bacterial RNAP that takes place away from the main nucleic acids binding channel and is independent of σ factor. The data also suggest that IsrA provides a scaffold for binding of a specific set of proteins, though whether this function is associated with its ability of binding RNAP or represents a separate function of IsrA is not yet clear and is discussed below.

**Figure 6.**
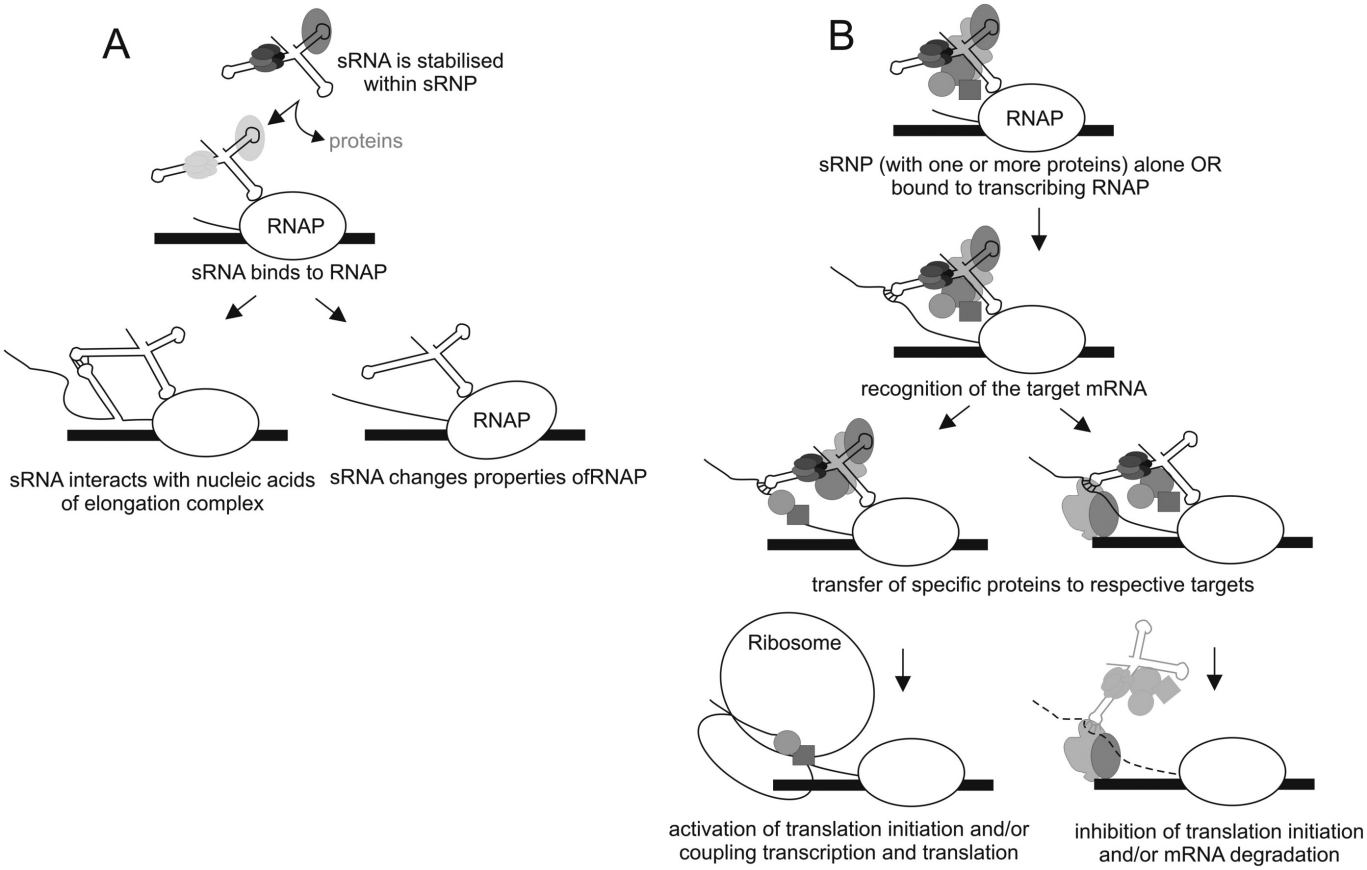
Hypothetical model of co-transcriptional functions of sRNPs formed by IsrA, and possibly other sRNAs. (**A**) Transcription regulation. IsrA stabilized by protein(s) such as Hfq and/or S1 binds specifically to core enzyme of RNAP via its stem 2, with or without the release of the proteins. During transcription elongation IsrA may be involved in regulation of transcription through interactions with nucleic acids of the elongation complex or by changing RNAP response to regulatory signals. (**B**) Translation regulation (note that shown is co-transcriptional regulation of translation, though our data do not exclude regulation that is independent of RNAP). sRNA associates with one or a set of proteins form sRNPs as directed by cellular requirements. Protein(s) of the sRNP remain silent until they are delivered to the target mRNA. The sRNP can associate with elongating RNAP (via stem 2 of IsrA), as suggested by our data, which would make its delivery to the target mRNA more efficient. Target mRNA is recognized via limited complementarity with the sRNA. Recognition of the target facilitates delivery of the protein(s) of the sRNP, which, depending on the set of proteins, determine the fate of the mRNA, such as activation or inhibition of translation or degradation of the mRNA. The sRNA can participate in the regulation of the mRNA along with the proteins or is released.

Whether IsrA acts as a transcriptional regulator *in vivo* remains to be seen. It is unlikely that it inhibits transcription in a manner 6S RNA does, as only non-specific binding of IsrA to RNAP, which does not happen *in vivo*, affects initiation (see the last section of the Results). Uniquely, specific binding of IsrA does not require σ factor (because it forms heparin-resistant complexes with core enzyme, and σ is not pulled-down with tagged IsrA from cells) and happens away from the main channel of RNAP (because IsrA binding is compatible with promoter and 6S RNA binding). On a conventional template (containing phage T7 promoter A1), specifically bound IsrA has no apparent effect on transcription (Figure 5A, lane 6 and B, lane 4). It is however possible that IsrA bound to initiating or elongating RNAP at a specific gene may directly affect transcription, for example, by base pairing with the nascent RNA or the DNA strands (Figure 6A). It is also possible that IsrA may participate in regulation of RNA dependent pausing and/or termination through interactions with RNA hairpins in the nascent RNA or affecting the conformational changes of RNAP on these signals (Figure 6A). Finally, as discussed below, IsrA can be delivered to mRNAs by RNAP to regulate their translation (Figure 6B).

The limited number of proteins that co-purified with IsrA indicates that this RNA forms particular sRNA-protein complexes. Therefore, another possible function of IsrA is bringing, via base pairing with the target RNA, proteins associated with it for regulation of translation and/or degradation of the target RNA (Figure 6B). We suggest that base-pairing interactions between target and regulatory RNAs might not be the main driving force for regulation of all these mRNAs. For example, stem 2 of IsrA has been shown to base-pair with the *csgD* mRNA, resulting in its translational down-regulation, while the sequence of the loop closing stem 3 mediates up-regulation of *flhDC* mRNA translation (21,22). The proposed mechanisms in which base-pair interactions between IsrA and the target mRNAs free or block the ribosome binding-site or start-codon, however, are poorly supported by phylogenetic evidence (Supplementary Figures S2 and S7). The involved seed-sequences in the mRNAs as well as in IsrA seem shorter than assumed, while extended base-pairing outwith the seed regions will be far from specific nor energetically favorable, because this would require disruption of a strong secondary structure in IsrA and a conserved stem-loop in *flhDC* mRNA. Moreover, as protein factors like CsrA, also bound by IsrA ((43); Figure 2A, Table 1), determine the half-life of this message (54), not only IsrA association will be relevant for its upregulation. The available data do not rule out that restricted base-pair interactions between the seed-regions actually function to enhance the local concentration of other factors brought by IsrA to the target RNA (Figure 6B). Even more intriguingly, sRNPs formed by IsrA (and possibly other sRNAs) could be delivered to mRNAs co-transcriptionally through interactions with the elongating RNAP (Figure 6B). Proteins, such as Hfq and S1, associated with IsrA were shown to influence transcription, and co-purified with RNAP (12,55–57), which could be mediated by sRNAs.

We propose that IsrA (and possibly other sRNAs) encounters its target mRNAs in the form of inactive RNPs, like in eukaryotic splicing, and deliver the needed factor(s) that only in the context of an ‘attenuation’ complex will be active and drive RNA transitions between translation-competent and/or translation-incompetent and/or degradation states, depending on the composition of the complex (Figure 6B). In this model, the protein composition of the sRNP determines the fate of the message, with the sRNA (partly) instrumental in delivering it. The activity of RNA-enzymes like PNPase or poly(A) polymerase PAP1 (low-represented in the cell; see below), could thereby be controlled by ‘storing’ these enzymes in the form of sRNPs in the cell so that their activity is only manifested when the sRNP is in a complex with a substrate, which may happen co-transcriptionally via interaction of IsrA with RNAP (Figure 6B).

For several sRNAs, like GcvB, MicA and RybB, a large number of possible mRNA-targets has been identified (58,59) while IsrA has been found to act, together with GlmZ, in a network of genes related to DNA repair (23), indicating a wider regulatory impact of many sRNAs than originally assumed. While we expected to find that Hfq and CsrA co-purify with IsrA based on the available data, some of the other proteins that were highly enriched in the IsrA-containing fraction came as a surprise to us. This was particularly true for proteins S1, ProQ and PNPase. Bacterial sRNAs are usually described as single agents with Hfq as a co-factor for stabilization, but our findings indicate the existence of various RNPs, consisting of IsrA and Hfq as a core, which have diversified by the binding of other proteins. Some of these, like RNAP, CsrA, S1 and ProQ can be expected to be primary IsrA-binders that would define particular sub-populations of IsrA sRNPs further specified by the association of secondary binding proteins. In this manner, IsrA could get involved in a multitude of processes. Note, that unlike the well-controlled assembly pathways needed to synthesize the well-defined eukaryotic snRNPs, our model allows for a more ‘fluid’ composition of bacterial sRNPs, depending on availability of factors and their interactions. Below we will discuss possible roles of the sRNPs formed by IsrA.

Association of protein S1, but not many of other 30S subunit proteins, suggests specific interaction of S1 with IsrA. The association of protein S1 with IsrA and other sRNAs (6S RNA, CsrB, DsrA, MicF, RyhB; (13)) could indicate that this protein, like Hfq, helps to stabilize sRNAs. Additionally, these sRNPs might contribute to translation initiation, for which 30S subunits – and thus S1- have to be on the mRNA before it is anchored by interactions with the Shine Dalgarno sequences and the start codon (60).

ProQ is another abundant protein that has been reported to associate with translating ribosomes (38) and is thought to require an RNA to upregulate translation of the transporter ProP that provides osmoprotection to cells (37). After the well-conserved Spot42 sRNA (13), IsrA would be the second candidate to facilitate ProQ control in the expression of ProP. Absence of ProQ inhibits biofilm formation (38), which relates to some of the roles reported for IsrA (43,53). Conservation within the *proP* 5′ UTR, is poor (Supplementary Figure S8), however, making ribo-regulation via sRNA base-pairing less likely.

Most intriguing was the co-purification of polynucleotide phosphorylase (PNPase) with IsrA (Figure 2), although this agrees with the finding that several other sRNAs rely for their stability on this protein (41). PNPase is a 3′ → 5′ exonuclease and part of the RNA degradosome (4) and could have been recruited via S1 (61) or Hfq, with which it forms a complex, together with the poly(A) polymerase PAP I (40). The very low abundant PAP I can interact with the helicase CsdA (45), which also functions in ‘cold-shock’ degradosomes (62). All these proteins co-purified with IsrA, which, when overexpressed, disrupt the synthesis of curli-fibers (an aspect of biofilm formation (21,22,53); Supplementary Figure S4). The mRNA for the transcription regulator of curli-synthesis, CsgD, is (one of many that are) upregulated in strains where PNPase or CsdA are absent (62). IsrA is assumed to induce the degradation of *csgD* mRNA (53), thus a sRNP consisting of IsrA, Hfq, S1, PAP I, PNPase, CsdA and possibly RNaseG (46,47,62,63) could be triggering *csgD* degradation by themselves or by providing the platform to recruit the remaining factors needed for this.

IsrA has two conserved GGA binding sites for the post-transcriptional regulator CsrA, which, for instance, inhibits translation of the *pgaABCD* mRNA and thereby the synthesis of an adhesin essential for biofilm formation. Suppression of *pgaABCD* mRNA translation was due to CsrA binding near the start codon or Shine-Dalgarno sequence (43) where a stem-loop (Supplementary Figure S7A) provides a high affinity CsrA binding site (64). Overexpression of IsrA, through its affinity for CsrA, counteracted this suppression, presumably by protein titration (43). Our results suggest a more specific mechanism, namely a direct transfer of CsrA from the mRNA to IsrA in the context of a sRNP-mRNA complex. A concomitant exchange with protein S1 or ProQ to protect the ribosome binding site of the *pgaABCD* mRNA, would prime this mRNA for translation (60). Post-transcriptional upregulation of the flagellar master regulator FlhDC by CsrA (42,54) could similarly be mediated by IsrA (22) albeit in reverse direction: IsrA delivering this protein when the *flhDC* message is encountered. GGA motifs recognized by CsrA occur frequently within Shine Dalgarno sequences or nearby start codons and a multitude of mRNAs – not only biofilm and motility genes – appear to be regulated by the binding of this protein (42). The above sketched scenario of protein exchange between target and sRNA within the context of a mRNA-sRNP complex could therefore provide a common regulatory model (Figure 6B).

## Supplementary Material

SUPPLEMENTARY DATA

## References

[1] Storz G., Vogel J., Wassarman K.M. (2011). Regulation by small RNAs in bacteria: expanding frontiers. Mol. Cell.

[2] De Lay N., Schu D.J., Gottesman S. (2013). Bacterial small RNA-based negative regulation: Hfq and its accomplices. J. Biol. Chem..

[3] Lalaouna D., Simoneau-Roy M., Lafontaine D., Massé E. (2013). Regulatory RNAs and target mRNA decay in prokaryotes. Biochim. Biophys. Acta.

[4] Bandyra K.J., Bouvier M., Carpousis A.J., Luisi B.F. (2013). The social fabric of the RNA degradosome. Biochim. Biophys. Acta.

[5] Alexander R.D., Innocente S.A., Barrass J.D., Beggs J.D. (2010). Splicing-dependent RNA polymerase pausing in yeast. Mol. Cell.

[6] Kos M., Tollervey D. (2010). Yeast pre-rRNA processing and modification occur cotranscriptionally. Mol. Cell.

[7] Aitken S., Alexander R.D., Beggs J.D. (2013). A rule-based kinetic model of RNA polymerase II C-terminal domain phosphorylation. J. R. Soc. Interface.

[8] Yanofsky C. (1981). Attenuation in the control of expression of bacterial operons. Nature.

[9] Richardson J.P. (1991). Preventing the synthesis of unused transcripts by Rho factor. Cell.

[10] Proshkin S., Rahmouni A.R., Mironov A., Nudler E. (2010). Cooperation between translating ribosomes and RNA polymerase in transcription elongation. Science.

[11] Larson M.H., Mooney R.A., Peters J.M., Windgassen T., Nayak D., Gross C.A., Block S.M., Greenleaf W.J., Landick R., Weissman J.S. (2014). A pause sequence enriched at translation start sites drives transcription dynamics in vivo. Science.

[12] Sukhodolets M.V., Garges S. (2003). Interaction of Escherichia coli RNA polymerase with the ribosomal protein S1 and the Sm-like ATPase Hfq. Biochemistry.

[13] Windbichler N., von Pelchrzim F., Mayer O., Csaszar E., Schroeder R. (2008). Isolation of small RNA-binding proteins from E. coli: evidence for frequent interaction of RNAs with RNA polymerase. RNA Biol..

[14] Lee D.J., Minchin S.D., Busby S.J.W. (2012). Activating transcription in bacteria. Annu. Rev. Microbiol..

[15] Cavanagh A.T., Sperger J.M., Wassarman K.M. (2012). Regulation of 6S RNA by pRNA synthesis is required for efficient recovery from stationary phase in E. coli and B. subtilis. Nucleic Acids Res..

[16] Kwek K.Y., Murphy S., Furger A., Thomas B., O'Gorman W., Kimura H., Proudfoot N.J., Akoulitchev A. (2002). U1 snRNA associates with TFIIH and regulates transcriptional initiation. Nat. Struct. Biol..

[17] Barrandon C., Spiluttini Bé., Bensaude O. (2008). Non-coding RNAs regulating the transcriptional machinery. Biol. Cell.

[18] Castelo-Branco G., Amaral P.P., Engström P. G., Robson S.C., Marques S.C., Bertone P., Kouzarides T. (2013). The non-coding snRNA 7SK controls transcriptional termination, poising, and bidirectionality in embryonic stem cells. Genome Biol..

[19] Ji X., Zhou Y., Pandit S., Huang J., Li H., Lin C.Y., Xiao R., Burge C.B., Fu X.-D. (2013). SR proteins collaborate with 7SK and promoter-associated nascent RNA to release paused polymerase. Cell.

[20] Chen S., Lesnik E.A., Hall T.A., Sampath R., Griffey R.H., Ecker D.J., Blyn L.B. (2002). A bioinformatics based approach to discover small RNA genes in the Escherichia coli genome. Bio Syst..

[21] Jørgensen M.G., Nielsen J.S., Boysen A., Franch T., Møller-Jensen J., Valentin-Hansen P. (2012). Small regulatory RNAs control the multi-cellular adhesive lifestyle of Escherichia coli. Mol. Microbiol..

[22] Thomason M.K., Fontaine F., De Lay N., Storz G. (2012). A small RNA that regulates motility and biofilm formation in response to changes in nutrient availability in Escherichia coli. Mol Microbiol.

[23] Modi S.R., Camacho D.M., Kohanski M.A., Walker G.C., Collins J.J. (2011). Functional characterization of bacterial sRNAs using a network biology approach. Proc. Natl. Acad. Sci. U.S.A..

[24] Athappily F.K., Hendrickson W.A. (1995). Structure of the biotinyl domain of acetyl-coenzyme A carboxylase determined by MAD phasing. Structure.

[25] Ulrich A., Andersen K.R., Schwartz T.U. (2012). Exponential megapriming PCR (EMP) cloning—seamless DNA insertion into any target plasmid without sequence constraints. PLoS ONE.

[26] Stringer A.M., Singh N., Yermakova A., Petrone B.L., Amarasinghe J.J., Reyes-Diaz L., Mantis N.J., Wade J.T. (2012). FRUIT, a scar-free system for targeted chromosomal mutagenesis, epitope tagging, and promoter replacement in Escherichia coli and Salmonella enterica. PLoS ONE.

[27] Datsenko K.A., Wanner B.L. (2000). One-step inactivation of chromosomal genes in Escherichia coli K-12 using PCR products. Proc. Natl. Acad. Sci. U.S.A..

[28] Castro-Roa D., Zenkin N. (2012). In vitro experimental system for analysis of transcription-translation coupling. Nucleic Acids Res..

[29] Budarina Z.I., Nikitin D.V., Zenkin N., Zakharova M., Semenova E., Shlyapnikov M.G., Rodikova E.A., Masyukova S., Ogarkov O., Baida G.E. (2004). A new Bacillus cereus DNA-binding protein, HlyIIR, negatively regulates expression of B. cereus haemolysin II. Microbiology.

[30] Gildehaus N., Neusser T., Wurm R., Wagner R. (2007). Studies on the function of the riboregulator 6S RNA from E. coli: RNA polymerase binding, inhibition of in vitro transcription and synthesis of RNA-directed de novo transcripts. Nucleic Acids Res..

[31] Klocko A.D., Wassarman K.M. (2009). 6S RNA binding to Eσ^70^ requires a positively charged surface of σ^70^ region 4.2. Mol. Microbiol..

[32] Dangerfield J.A., Windbichler N., Salmons B., Günzburg W.H., Schröder R. (2006). Enhancement of the StreptoTag method for isolation of endogenously expressed proteins with complex RNA binding targets. Electrophoresis.

[33] Bachler M., Schroeder R., von Ahsen U. (1999). StreptoTag: a novel method for the isolation of RNA-binding proteins. RNA.

[34] Windbichler N., Schroeder R. (2006). Isolation of specific RNA-binding proteins using the streptomycin-binding RNA aptamer. Nat. Protoc..

[35] Wassarman K.M., Saecker R.M. (2006). Synthesis-mediated release of a small RNA inhibitor of RNA polymerase. Science.

[36] Wilusz C.J., Wilusz J. (2013). Lsm proteins and Hfq: Life at the 3′ end. RNA Biol..

[37] Chaulk S.G., Smith Frieday M.N., Arthur D.C., Culham D.E., Edwards R.A., Soo P., Frost L.S., Keates R.A.B., Glover J.N.M., Wood J.M. (2011). ProQ is an RNA chaperone that controls ProP levels in Escherichia coli. Biochemistry.

[38] Sheidy D.T., Zielke R.A. (2013). Analysis and Expansion of the Role of the Escherichia coli Protein ProQ. PLoS ONE.

[39] Lauber M.A., Rappsilber J., Reilly J.P. (2012). Dynamics of ribosomal protein S1 on a bacterial ribosome with cross-linking and mass spectrometry. Mol. Cell Proteomics.

[40] Mohanty B.K., Maples V.F., Kushner S.R. (2004). The Sm-like protein Hfq regulates polyadenylation dependent mRNA decay in Escherichia coli. Mol. Microbiol..

[41] De Lay N., Gottesman S. (2011). Role of polynucleotide phosphorylase in sRNA function in Escherichia coli. RNA.

[42] Romeo T., Vakulskas C.A., Babitzke P. (2013). Post-transcriptional regulation on a global scale: form and function of Csr/Rsm systems. Environ. Microbiol..

[43] Jørgensen M.G., Thomason M.K., Havelund J., Valentin-Hansen P., Storz G. (2013). Dual function of the McaS small RNA in controlling biofilm formation. Genes Dev..

[44] Bishop A.C., Xu J., Johnson R.C., Schimmel P., de Crécy-Lagard V. (2002). Identification of the tRNA-dihydrouridine synthase family. J. Biol. Chem..

[45] Raynal L.C., Carpousis A.J. (1999). Poly(A) polymerase I of Escherichia coli: characterization of the catalytic domain, an RNA binding site and regions for the interaction with proteins involved in mRNA degradation. Mol. Microbiol..

[46] Mohanty B.K., Kushner S.R. (2011). Bacterial/archaeal/organellar polyadenylation. Wiley Interdiscip. Rev. RNA.

[47] Lee K., Bernstein J.A., Cohen S.N. (2002). RNase G complementation of rne null mutation identifies functional interrelationships with RNase E in Escherichia coli. Mol. Microbiol..

[48] Otaka H., Ishikawa H., Morita T., Aiba H. (2011). PolyU tail of rho-independent terminator of bacterial small RNAs is essential for Hfq action. Proc. Natl. Acad. Sci. U.S.A..

[49] Sauer E., Schmidt S., Weichenrieder O. (2012). Small RNA binding to the lateral surface of Hfq hexamers and structural rearrangements upon mRNA target recognition. Proc. Natl. Acad. Sci. U.S.A..

[50] Vecerek B., Rajkowitsch L., Sonnleitner E., Schroeder R., Bläsi U. (2008). The C-terminal domain of Escherichia coli Hfq is required for regulation. Nucleic Acids Res..

[51] Olsen A.S., Møller-Jensen J., Brennan R.G., Valentin-Hansen P. (2010). C-terminally truncated derivatives of Escherichia coli Hfq are proficient in riboregulation. J. Mol. Biol..

[52] Dimastrogiovanni D., Fröhlich K.S., Bandyra K.J., Bruce H.A., Hohensee S., Vogel J., Luisi B.F. (2014). Recognition of the small regulatory RNA RydC by the bacterial Hfq protein. Elife.

[53] Mika F., Hengge R. (2013). Small Regulatory RNAs in the Control of Motility and Biofilm Formation in E. coli and Salmonella. Int. J. Mol. Sci..

[54] Yakhnin A.V., Baker C.S., Vakulskas C.A., Yakhnin H., Berezin I., Romeo T., Babitzke P. (2013). CsrA activates flhDC expression by protecting flhDC mRNA from RNase E-mediated cleavage. Mol. Microbiol..

[55] Sukhodolets M.V., Garges S., Adhya S. (2006). Ribosomal protein S1 promotes transcriptional cycling. RNA.

[56] Guisbert E., Rhodius V.A., Ahuja N., Witkin E., Gross C.A. (2007). Hfq modulates the σ^E^-mediated envelope stress response and the σ^32^-mediated cytoplasmic stress response in Escherichia coli. J. Bacteriol..

[57] Lorenz C., Gesell T., Zimmermann B., Schoeberl U., Bilusic I., Rajkowitsch L., Waldsich C., von Haeseler A., Schroeder R. (2010). Genomic SELEX for Hfq-binding RNAs identifies genomic aptamers predominantly in antisense transcripts. Nucleic Acids Res..

[58] Pulvermacher S.C., Stauffer L.T., Stauffer G.V. (2009). Role of the sRNA GcvB in regulation of cycA in Escherichia coli. Microbiology.

[59] Gogol E.B., Rhodius V.A., Papenfort K., Vogel J., Gross C.A. (2011). Small RNAs endow a transcriptional activator with essential repressor functions for single-tier control of a global stress regulon. Proc. Natl. Acad. Sci. U.S.A..

[60] de Smit M.H., van Duin J. (2003). Translational standby sites: how ribosomes may deal with the rapid folding kinetics of mRNA. J. Mol. Biol..

[61] Feng Y., Huang H., Liao J., Cohen S.N. (2001). Escherichia coli poly(A)-binding proteins that interact with components of degradosomes or impede RNA decay mediated by polynucleotide phosphorylase and RNase E. J. Biol. Chem..

[62] Phadtare S. (2012). Escherichia coli cold-shock gene profiles in response to over-expression/deletion of CsdA, RNase R and PNPase and relevance to low-temperature RNA metabolism. Genes Cells.

[63] Umitsuki G., Wachi M., Takada A., Hikichi T., Nagai K. (2001). Involvement of RNase G in in vivo mRNA metabolism in Escherichia coli. Genes Cells.

[64] Dubey A.K., Baker C.S., Romeo T., Babitzke P. (2005). RNA sequence and secondary structure participate in high-affinity CsrA-RNA interaction. RNA.

