# Supplementary material for: Ribonucleoprotein particles of bacterial small non-coding RNA IsrA (IS61 or McaS) and its interaction with RNA polymerase core may link transcription to mRNA fate
Source: Nucleic Acids Res. 2015 Nov 24;44(6):2577–92. doi: 10.1093/nar/gkv1302 (PMC4824073; doi:10.1093/nar/gkv1302)
Supplement: SUPPLEMENTARY DATA [file supp_44_6_2577__index.html]

Ribonucleoprotein particles of bacterial small non-coding RNA IsrA (IS61 or McaS) and its interaction with RNA polymerase core may link transcription to mRNA fate — SUPPLEMENTARY DATA 

# Ribonucleoprotein particles of bacterial small non-coding RNA IsrA (IS61 or McaS) and its interaction with RNA polymerase core may link transcription to mRNA fate

## SUPPLEMENTARY DATA

- SUPPLEMENTARY DATA
