# Supplementary material for: Ribonucleoprotein particles of bacterial small non-coding RNA IsrA (IS61 or McaS) and its interaction with RNA polymerase core may link transcription to mRNA fate
Source: Nucleic Acids Res. 2015 Nov 24;44(6):2577–92. doi: 10.1093/nar/gkv1302 (PMC4824073; doi:10.1093/nar/gkv1302)
Supplement: SUPPLEMENTARY DATA [file supp_gkv1302_vanNues-Zenkin-Suppl-corrected-refs2.pdf]

Supplementary materials for

Ribonucleoprotein particles of bacterial small non-coding RNA IsrA (IS61 or McaS) and its interaction with RNA polymerase core may link transcription to mRNA fate

Rob W. van Nues, Daniel Castro-Roa, Yulia Yuzenkova and Nikolay Zenkin*

Centre for Bacterial Cell Biology, Institute for Cell and Molecular Biosciences, Newcastle University, Newcastle upon Tyne, UK

* To whom correspondence should be addressed. Tel: 44 1912083227; Fax: 44 1912083205; Email: n.zenkin@newcastle.ac.uk

Running title; sRNPs interacting with RNA polymerase core
Keywords: small RNA/ribonucleoprotein particle/IsrA/RNA polymerase/core enzyme

SUPPLEMENTARY TEXT

IsrA expression

IsrA was fully expressed during the mid-logarithmic growth phase (OD600 of 1.5-1.9) in RL based strains (Supplementary Table), with levels being much lower at OD600 > 2.4, which is different from MG1655, where IsrA expression peaks at an OD600 of 2.5 during the transition to stationary growth (4). The RL$^{\textit{rpo}C\mathrm{HIS}}$ strain routinely used at the time in our lab for RNAP purification, and its parent, RL, have a different genetic background than the commonly used MG1655 (Supplementary Table). We observed a marked difference in the expression levels of IsrA between these strains or their derivatives (cf. Fig. 1E, lanes 3 and 2, 5, 6) during mid-log phase. Also a strain constructed from the original JC7623, the parental strain of RL324 obtained from a stock center (Supplementary Table) expressed very low levels of IsrA, undetectable by Northern blotting in total lysates from cells harvested at an OD600 of 1.5-1.9 (Fig. 1E, lane 4). A reason for this difference in expression, as found by sequencing of the *isrA*-locus, could be a single mutation in the -10 region that created an optimal promoter for RNAP: TATAAT in the RL324-based strains instead of TATAAC in MG1655 and JC7623 (Supplementary Fig. S1F). Still, although minor amounts were detected, IsrA co-purifed with RNAP from MG1655 and JC7623 cells (Fig. 1E, lanes 9, 10), demonstrating that the association of this sRNA with RNAP is strain-independent.

Supplementary Table. Strains used

Strain	*Genotype*	*Reference or Source*
BW25113	F^{-}, *DE(araD-araB)567, lacZ4787(del)::rrnB-3, LAM^{-}, rph-1, DE(rhaD-rhaB)568, hsdR514*	Datsenko and Wanner (2000) (1)
MG1655	F-, lambda-, *rph-1*	Robert Landick
JC7623	*thr-1, ara-14, leuB6, Δ(gpt-proA)62, lacY1, tsx-33, supE44, galK2, rac^{-}, hisG4(Oc), rfbD1, mgl-51, rpsL31, kdgK51, xyl-5, mtl-1, argE3 (Oc), thi-1, qsr^{-}, sbcC201, recB21, recC22, sbcB15, rpoS396(Am)*	Horii and Clark (1973) (2) Visick and Clarke (1997) (3) Coli Genetic Stock Center (cgsc.biology.yale.edu)
RL	also RL324; is JC7623, G1405758 → A (MG1655 numbering)	Robert Landick
RL$^{rpo\text{CHIS}}$	also RL721; is RL, rpoC3531(His6) zja::kan	Robert Landick
MG1655$^{rpo\text{CHIS}}$	MG1655, rpoC(His6)::kan	Rachel Mooney, Robert Landick
BW-62; BW-74	BW25113, rpoC(3C-BCCP)::kan	λRed recombinase integrant
MG1655$^{rpo\text{CBCCP}}$	MG1655, rpoC(3C-BCCP)::kan	P1 transductant from BW-62
JC7623$^{rpo\text{CBCCP}}$	JC7623, rpoC(3C-BCCP)::kan	P1 transductant from BW-74
RL$^{rpo\text{CBCCP}}$	RL, rpoC(3C-BCCP)::kan	P1 transductant from BW-74
RLΔ*isrA*	RL, ΔisrA::cat	λRed recombinase integrant
RLΔ*hfq*	RL, Δhfq::cat	λRed recombinase integrant
RL*hfq65*	RL, hfq65::cat	λRed recombinase integrant
RL$^{rpo\text{CBCCP}}$Δ*isrA*	RLΔ*isrA*, rpoC(3C-BCCP)::kan	P1 transductant from BW-62
RL$^{rpo\text{CBCCP}}$*hfq65*	RL*hfq65*, rpoC(3C-BCCP)::kan	P1 transductant from BW-62
RLΔ*isrA*-p*isrA*	RLΔ*isrA*, pGemT-*isrA*	RLΔ*isrA* transformant
RL$^{rpo\text{CBCCP}}$Δ*isrA*, pGemT	RL$^{rpo\text{CBCCP}}$Δ*isrA*, pGemT (vector)	RL$^{rpo\text{CBCCP}}$Δ*isrA* transformant (cf. Suppl. Fig. 1F)
RL$^{rpo\text{CBCCP}}$Δ*isrA*-p*isrA*$^{\Delta B}$	RL$^{rpo\text{CBCCP}}$Δ*isrA*, pGemT-*isrA*ΔB	
RL$^{rpo\text{CBCCP}}$Δ*isrA*-p*isrA*$^{i\Delta 2}$	RL$^{rpo\text{CBCCP}}$Δ*isrA*, pGemT-*isrA*iΔ2	

Strain	*Genotype*	*Reference or Source*
RL$^{rpo\mathrm{CBCCP}}$Δ*isrA*-p*isrA*^{i2loop}	RL$^{rpo\mathrm{CBCCP}}$Δ*isrA*, pGemT-*isrA*i2loop	RL$^{rpo\mathrm{CBCCP}}$Δ*isrA* transformant (cf. Suppl. Fig. 1F)
RL$^{rpo\mathrm{CBCCP}}$Δ*isrA*-p*isrA*^{3tag}	RL$^{rpo\mathrm{CBCCP}}$Δ*isrA*, pGemT-*isrA3tag*	

Supplementary Figure S1. Schematic overview of recombinant strains and plasmids.

(A) Peptide sequence of the C-terminal region of RpoC with attached the C-terminal region of BCCP (yellow, encoded by nucleotides 211-468 of *accB)* that will be biotinylated on the boxed lysine. RNAP can be released from streptavidin sepharose by HRV3C cleavage of LEVLFQ/GP (red).

(B) Corresponding nucleotide sequence (top) and schematic overview of the integration cassette.

(C) Peptide sequence of Hfq and hfq65.

(D) Schematic of modifying the *hfq* locus by insertion of a stop-codon linked to the *cat* marker.

(E) Overview of the *isrA* gene disruption and

(F) of IsrA mutants constructed in pGemT Easy within their genomic context (from -205 to +42).

The -10 region of *isrA* in RL$^{rpoC\mathrm{HIS}}$ and RL contains a T which is a C in MG1655 and JC7623 (red arrow). IsrA (mutants) were detected by Northern hybridization with a ^{32}P-radioactively end-labeled oligo complementary to nt 4-26 (maroon arrow). Integration cassettes were amplified with primers indicated by purple arrows, and their insertion verified by colony PCR with primers shown as black arrows. Relevant nucleotide positions are given with respect to the start of the coding regions as available via http://ecocyc.org. The numbering of downstream positions is with respect to the end of the coding regions and include the stop codons (asterisk). The genes providing resistance to kanamycin (*kan*, cloned length is within parentheses), or chloramphenicol (*cat*), and the c1 primer are described in (1).

Supplementary Figure S2. Phylogenetic analysis of genes encoding IsrA homologs.

(A) Alignment of promoter regions. The -35 and -10 elements in *E. coli* are indicated, as well as the identified CRP element (4). The nucleotide in the -10 box that is mutated in RL-based strains (to a T), is indicated (red arrow).

(B) Alignment of IsrA genes with the resulting sequence logo at the top. Brackets indicate paired nucleotides according to mfold or according to phylogenetic comparison. Protein binding sites (CsrA) or regions implied in translational regulation of *csgD* or *flhDC* mRNAs are indicated and mostly well-conserved.

(C) Consensus secondary structure predicted on the basis of the alignment in (B) and obtained as described in the Materials and Methods.

(D) Variation in the genomic organization of the *isrA* locus in bacterial species related to *E. coli.*

Supplementary Figure S3. Control to pull-down experiment of Figure 2.

Streptotag of IsrA does not affect its association with RNAP *in vivo*. Northern blot analysis of RNAs that co-purified with RNAP from strain RL$^{rpoC\mathrm{BCCP}}$Δ*isrA* in which isrA3-tag was expressed.

Supplementary Figure S4. Curli synthesis is not affected by IsrA overexpression after truncation of Hfq.

Congo-red plate-assay with isogenic strains (expressing RNAP with a biotinylated tag, RL$^{rpoC\mathrm{BCCP}}$) used in the co-purification experiment (Figs. 1 D, E). Due to a promoter mutation, the parental strain (wt) overexpresses IsrA, which affects curli synthesis as indicated by reduced red-pigment formation (4, 5). In the absence of IsrA (Δ*isrA*) or when a truncated version of Hfq (containing the first 65 amino acids and lacking the C-terminal tail; Supplementary Fig. S1C) is expressed (*hfq65*), curli-fibers are stainable. The right panels show a serial dilution series (and thereby cells at various growth phases), the left panels independently obtained duplicates of the indicated strains, uniformly spread as large spots. The bottom panel shows the scans before cleanup of surrounding background and digital enhancement, the results of which are shown at the top to better visualize the differences between the strains.

Supplementary Figure S5. Competitor RNAs.

Unlabeled competitor RNAs, separated on 10% PAGE and stained with methylene blue (left) or ethidium bromide (right).

Supplementary Figure S6. Controls to pull-down experiment of Figure 4D.

(A) Secondary structure model of IsrA with altered regions indicated. In IsrA-mutant $isrA^{i2loop}$ (i2loop) the loop closing stem 2 has been replaced with GAAG, whereas in mutants $isrA^{\Delta B}$ (ΔB) and $isrA^{i\Delta 2}$ (iΔ2) the regions in blue and red, respectively, were deleted (see Supplementary Fig. S1F).

(B) Plasmids from which the mutant IsrA RNAs were expressed, along with an empty vector (pGemT), were transformed into strain RL$^{rpoC\mathrm{BCCP}}$Δ*isrA* with a disrupted IsrA gene and expressing RNAP with a biotinylated tag that can be removed by HRV3C (3C) protease (Supplementary Table and Supplementary Figs. S1A, 1E). A strain expressing RNAP without the biotinylated tag (RLΔ*isrA*-p*isrA*) was used as a control. Proteins (and associated sRNAs; see Fig. 2, Table 1) were released from the beads with 3C and analyzed by SDS/PAGE on a 4-20% gradient gel.

Supplementary Figure S7. Phylogenetic analysis of IsrA target mRNAs.

Alignment of 5' UTRs of *pgaABCD* (A), *flhDC* (B) and *csgD* (C) with indicated the transcription start site (gray arrow), ribosome binding site (SD), start codon (ATG). Secondary structure elements that are supported by phylogenetic evidence (i.e. compensatory nucleotide changes) are marked by brackets (with in red a stem-loop interfering with the model proposed by (6)). Regions of base pairing interactions with sRNAs IsrA, ArcZ, OxyS, OmrA/OmrB, GcvB and RprA that have been tested by compensatory base pair changes (apart from GcvB) are boxed (as reviewed in (6)). The names of downregulating sRNAs are in red. Asterisks preceding the names for strains on the left indicate that IsrA was not found in the genome of these strains (cf. Supplementary Figure S2D).

Supplementary Figure S8. Phylogenetic analysis of ProP UTRs.

Annotations as in Supplementary Figure S7. Stop codons (*) and putative terminating hair-pins are indicated.

Supplementary References

1. Datsenko, K. A. & Wanner, B. L. (2000) One-step inactivation of chromosomal genes in Escherichia coli K-12~using PCR products *PNAS*, **97**, 6640-6645.

2. Horii, Z. & Clark, A. J. (1973) Genetic analysis of the recF pathway to genetic recombination in Escherichia coli K12: isolation and characterization of mutants. *J Mol Biol*, **80**, 327-344.

3. Visick, J. E. & Clarke, S. (1997) RpoS- and OxyR-independent induction of HPI catalase at stationary phase in Escherichia coli and identification of rpoS mutations in common laboratory strains. *J Bacteriol*, **179**, 4158-4163.

4. Thomason, M. K.; Fontaine, F.; De Lay, N. & Storz, G. (2012) A small RNA that regulates motility and biofilm formation in response to changes in nutrient availability in Escherichia coli. *Mol Microbiol*, **84**, 17-35.

5. Jørgensen, M. G.; Nielsen, J. S.; Boysen, A.; Franch, T.; Møller-Jensen, J. & Valentin-Hansen, P. (2012) Small regulatory RNAs control the multi-cellular adhesive lifestyle of Escherichia coli. *Mol Microbiol*, **84**, 36-50.

6. Mika, F. & Hengge, R. (2013) Small Regulatory RNAs in the Control of Motility and Biofilm Formation in E. coli and Salmonella. *Int J Mol Sci*, **14**, 4560-79.

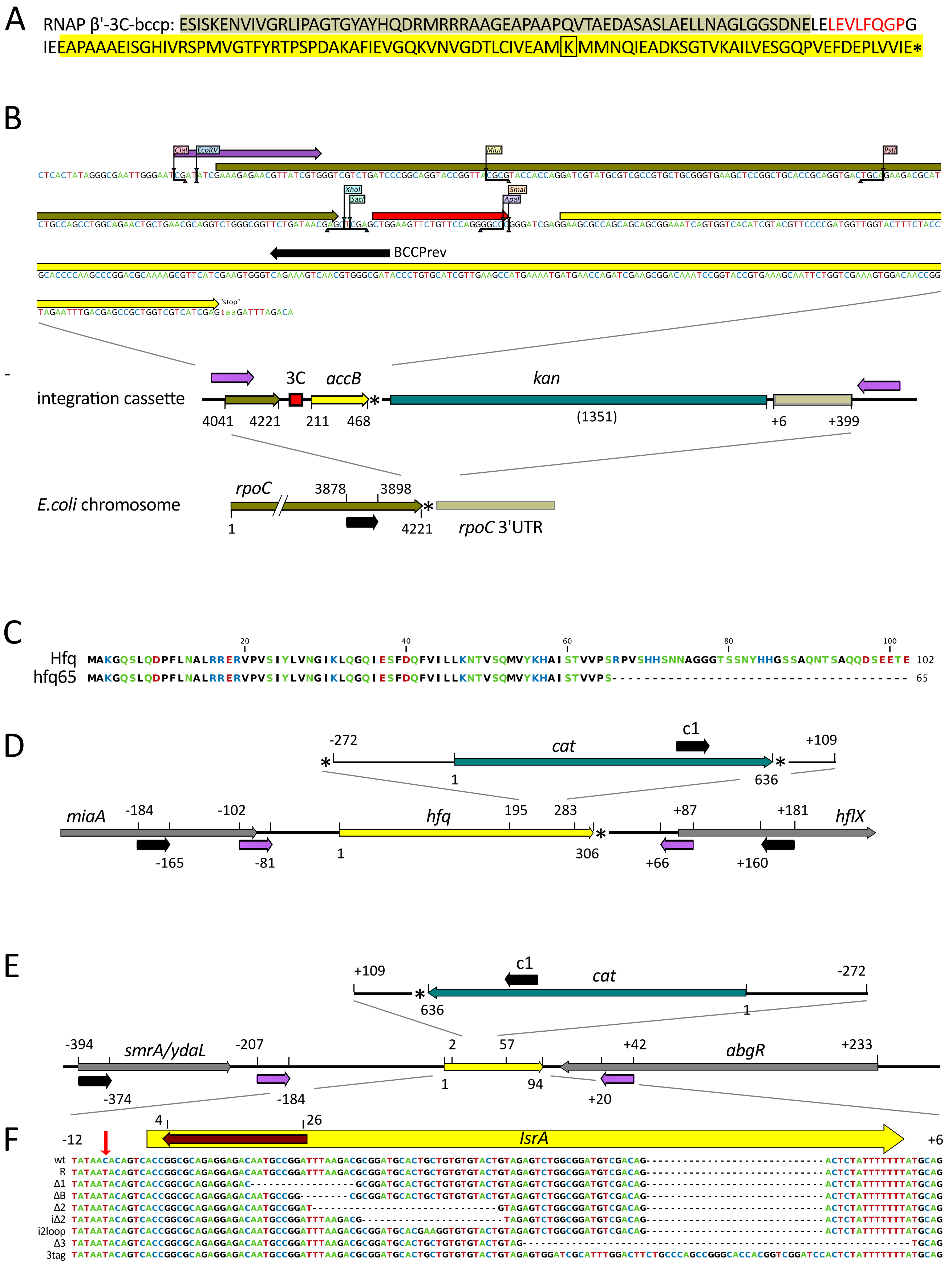

Van Nues et al.
Supplementary Figure S1

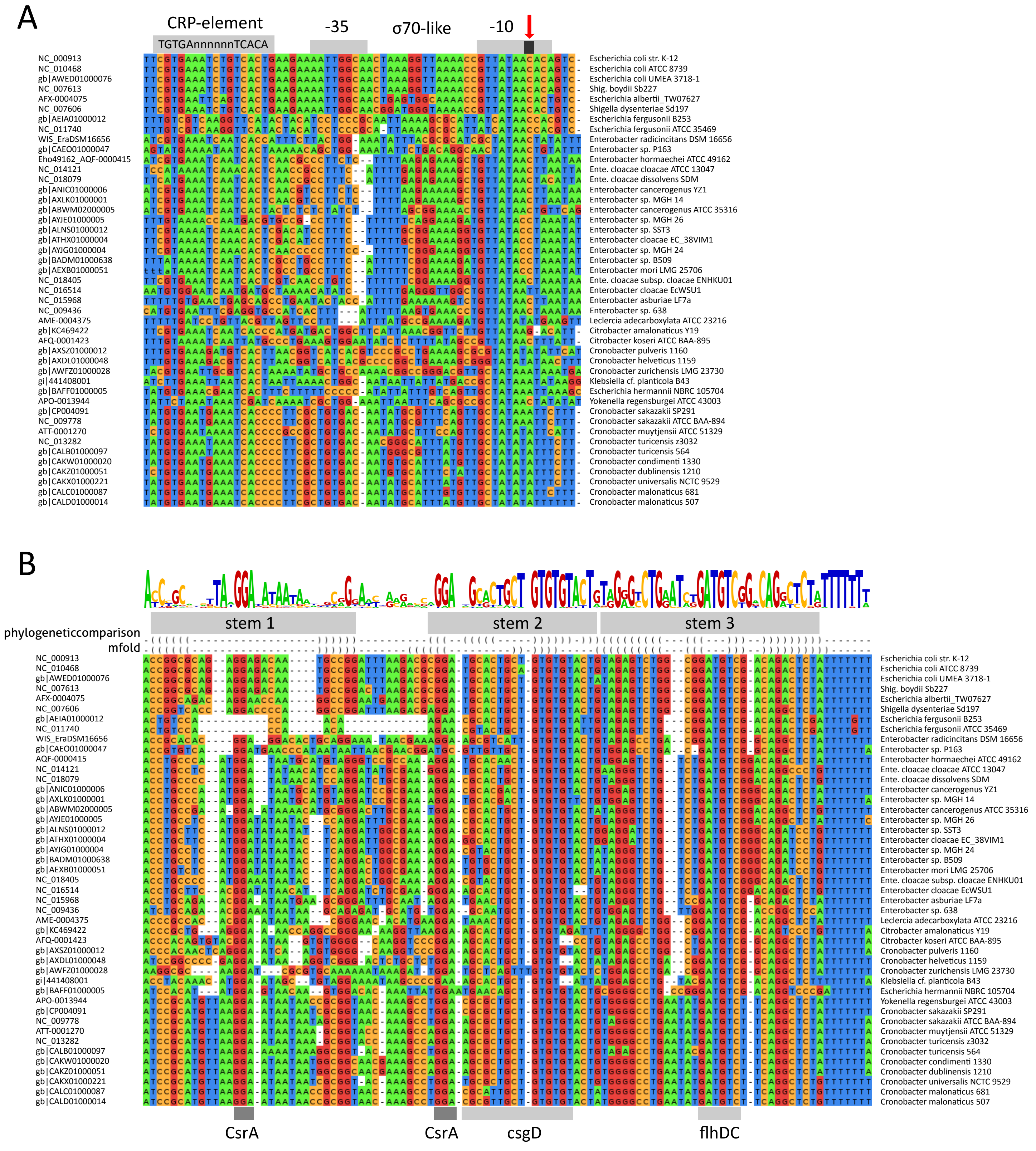

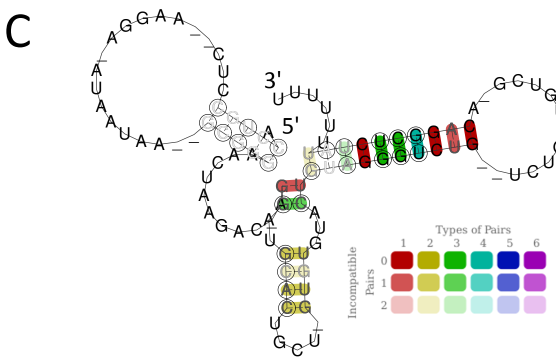

D

	ydaL	*isrA*	*abgR*
Enterobacter mori LMG 25706	+	+	+
Citrobacter koseri ATCC BAA-895	+	+	-
Enterobacter aerogenes EA1509E	+	-	+
Enterobacter sp. Ag1	+	-	+
Klebsiella oxytoca KCTC 1686	+	-	+
Citrobacter freundii ballerup 7851	+	-	-
Citrobacter rodentium ICC168	+	-	-
Citrobacter youngae ATCC 29220	+	-	-
Cedecea davisae ATCC 33431	+	-	-
Salmonella typh. SL1344	+	-	-
Salmonella paratyphi B SPB7	+	-	-

Van Nues et al.
Supplementary Figure S2

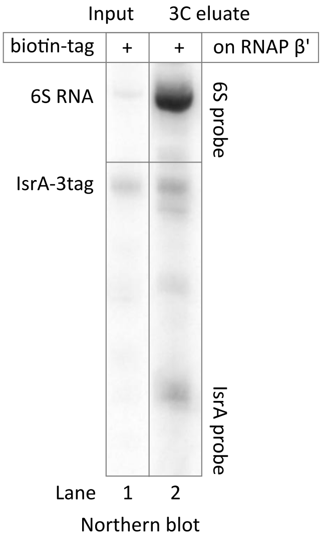

Van Nues et al.
Supplementary Figure S3

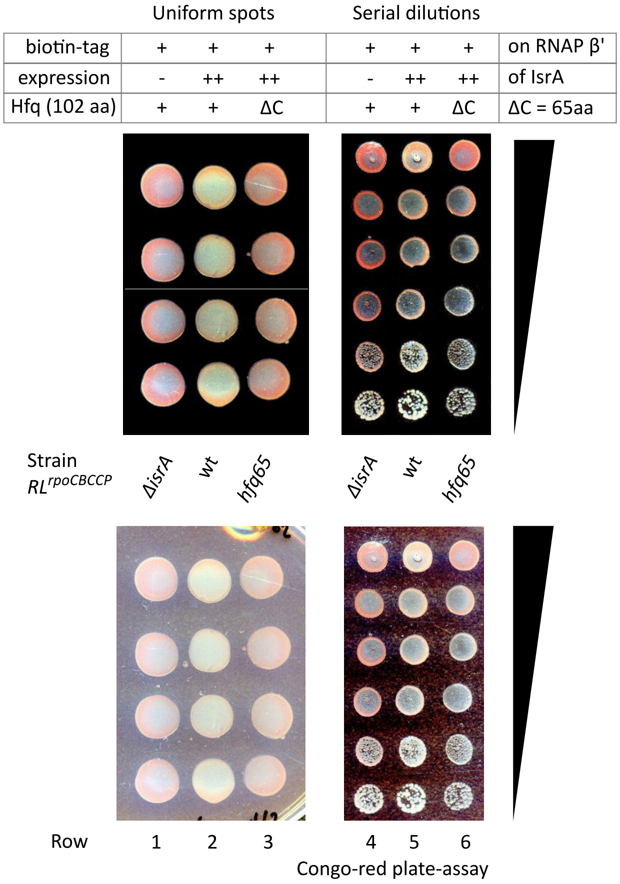

Van Nues et al.
Supplementary Figure S4

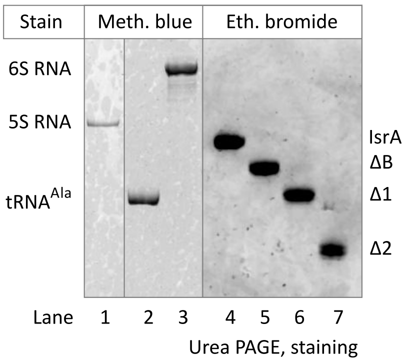

Van Nues et al.
Supplementary Figure S5

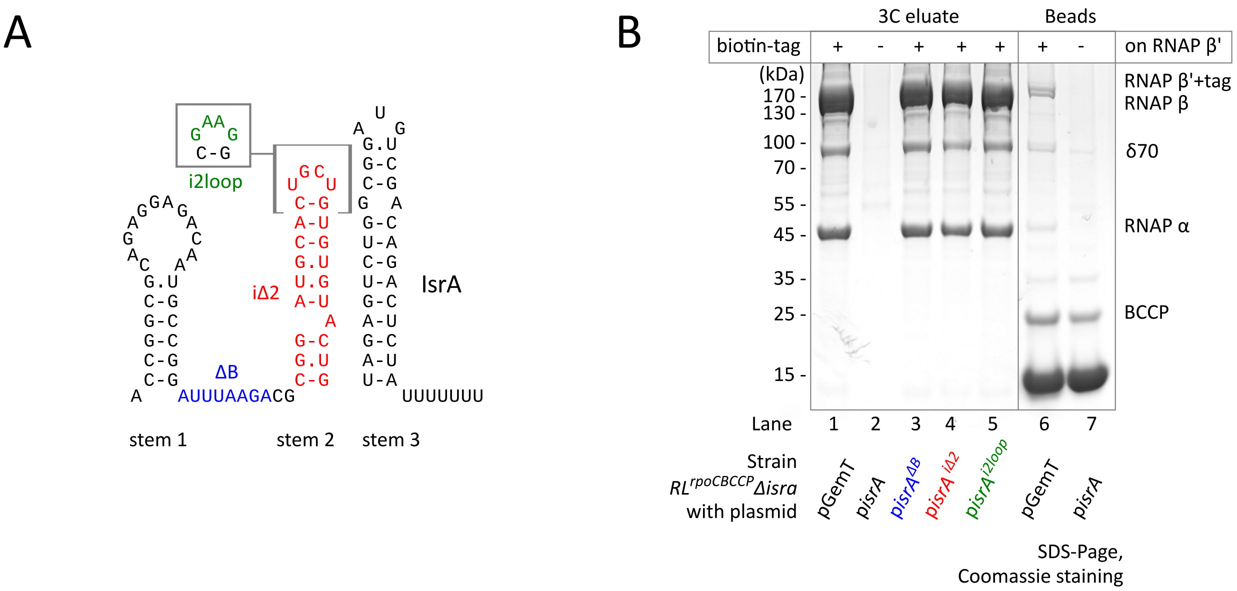

Van Nues et al.
Supplementary Figure S6

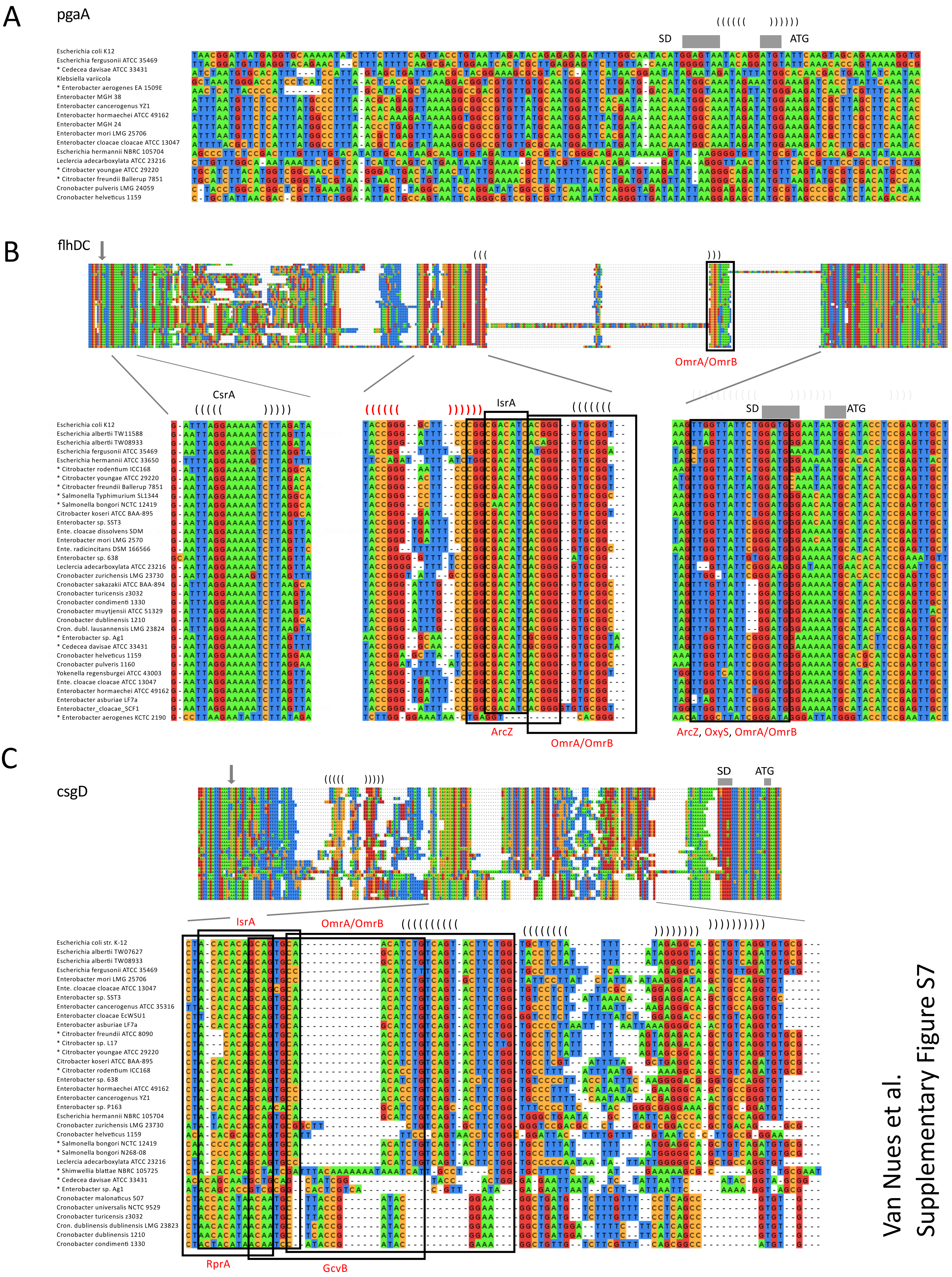

Van Nues et al.
Supplementary Figure S7

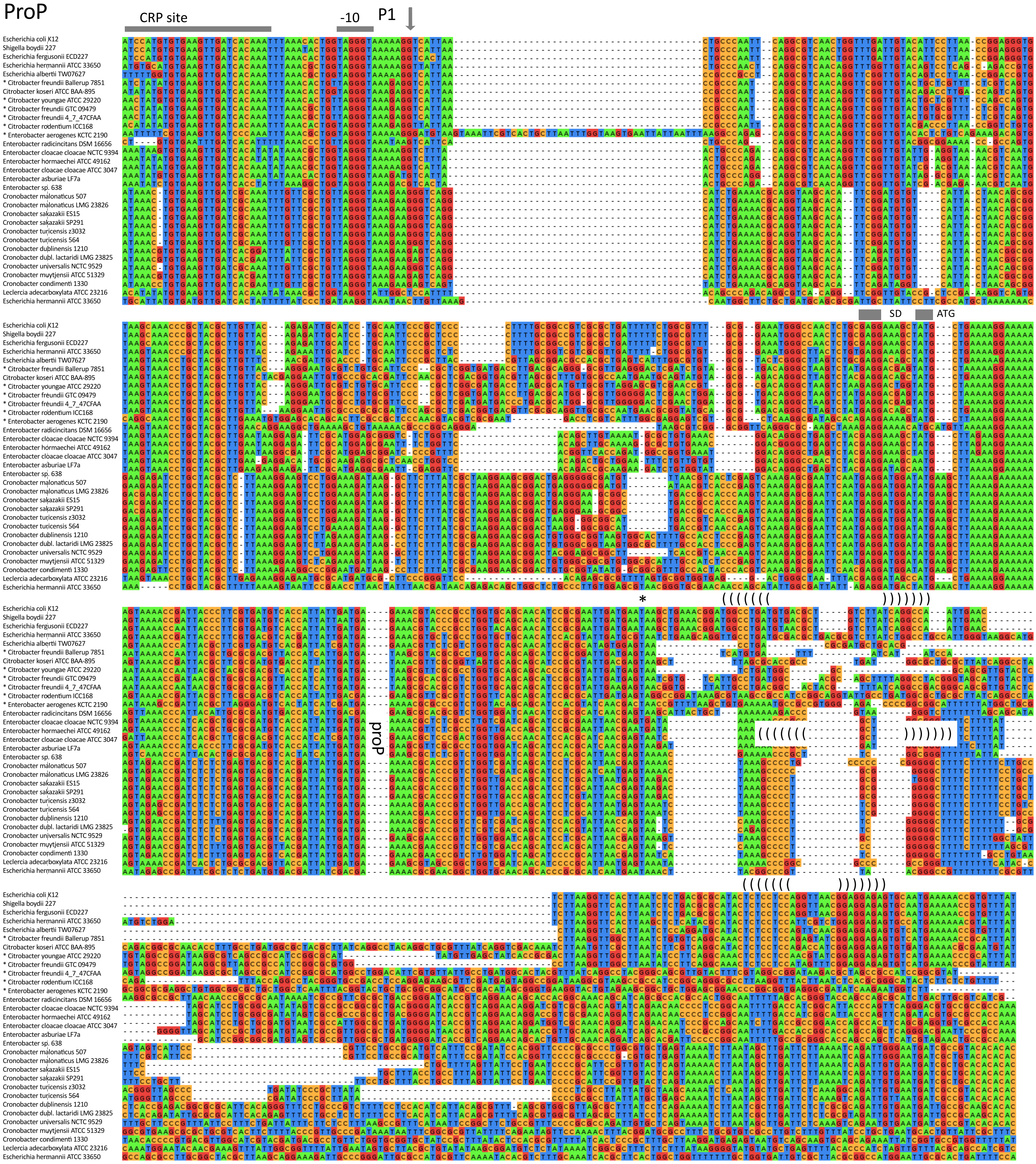

Van Nues et al.
Supplementary Figure S8

Supplementary data

isrA3tag associated proteins

Page 'Data3analyses' combines the three datasets (GPM-265, GPM-264 and GPM-266) on the subsequent pages

Data3analyses

Accession	UniProt	Ecoli	gene	protein	Mr	log(e)	#	Description
gi|16128878	P0AG67	b0911	rpsA	S1	61.1	-299.83	125	30S ribosomal subunit protein S1
gi|16131994	P0A6X3	b4172	hfq	Hfq	11.2	-296.60	362	global sRNA chaperone; host factor for RNA phage Q beta replication
gi|49176156	P45577	b1831	proQ	ProQ	25.9	-238.23	128	RNA chaperone; probable regulator of ProP translation.
gi|145698316	P05055	b3164	pnp	PNPase	77.1	-130.10	44	polynucleotide phosphorylase; polynucleotide polymerase.
gi|16131817	P0A8V2	b3987	rpoB	RNAP β	150.5	-106.33	33	RNA polymerase; beta subunit; in L1 translationally controlled operon with rplA (L1), rplK, rplL, rplJ, rpoB, rpoC
gi|16131174	P0A7Z4	b3295	rpoA	RNAp α	36.5	-66.87	28	RNA polymerase; alpha subunit; in rpsD translationally controlled operon
gi|16131602	P0ABB0	b3734	atpA	AtpA	55.2	-66.33	23	F1 sector of membrane-bound ATP synthase
gi|90111550	P0A9P6	b3162	deaD	CsdA	70.5	-65.80	22	ATP-dependent RNA helicase; can replace RlhE in (cold-shock) degradosome; implicated in ribosome biogenesis
gi|16130603	P69913	b2696	csrA	CsrA	6.9	-57.03	38	pleiotropic regulatory protein for carbon source metabolism.
gi|16131818	P0A8T7	b3988	rpoC	RNAP β'	155.1	-51.13	19	RNA polymerase; beta prime subunit; in L1 translationally controlled operon with rplA (L1), rplK, rplL, rplJ, rpoB, rpoC
gi|145698340	P32695	b4049	dusA	DusA	36.8	-42.73	16	tRNA-dihydrouridine synthase A (with tRNA-serV??)
gi|16128424	P0A9M0	b0439	lon	Lon	87.4	-41.20	17	DNA-binding ATP-dependent protease La.
gi|90111563	P0A9J0	b3247	rng	RNAseG	55.3	-12.93	7	ribonuclease G.
gi|162135892	P0ABF1	b0143	pcnB	PAP I	53.8	-12.40	9	poly(A) polymerase.
gi|49176463	P0ADB7	b4411	encB	EncB	4.8	-12.40	3	entericidin B membrane lipoprotein; translationally repressed by MicA; Component of entericidin toxin-antitoxin complex
gi|90111588	P46837	b3407	yhgF	YhgF	85.1	-11.90	4	yhgF; has C-terminal S1; predicted transcriptional accessory protein
gi|16131143	P0ABD8	b3255	accB	BCCP	16.7	-9.23	4	acetyl CoA carboxylase; BCCP subunit; C-terminal region used as tag on RNAP β'
gi|16131220	P02359	b3341	rpsG	S7	20	-73.23	30	30S ribosomal subunit protein S7; in operon with rpsL (S12)
gi|16131814	P0A7L0	b3984	rplA	L1	24.7	-62.23	26	provides translation regulation of L1-L11 (rplAK) part of the operon upstream of rpoB-rpoC; 50S ribosomal subunit protein L1
gi|16131193	P0A7V3	b3314	rpsC	S3	26	-50.23	22	30S ribosomal subunit protein S3.
gi|16131182	P0A7W1	b3303	rpsE	S5	17.6	-40.90	17	30S ribosomal subunit protein S5.
gi|16131177	P0A7S9	b3298	rpsM	S13	13.1	-40.80	20	30S ribosomal subunit protein S13; in rpsD translationally controlled operon
gi|16131120	P0A7X3	b3230	rpsI	S9	14.8	-40.17	19	30S ribosomal subunit protein S9.
gi|16131175	P0A7V8	b3296	rpsD	S4	23.5	-39.40	24	30S ribosomal subunit protein S4; in rpsD translationally controlled operon
gi|16132025	P0A7R1	b4203	rplI	L9	15.8	-36.17	13	50S ribosomal subunit protein L9.
gi|16131180	P02413	b3301	rplO	L15	15	-35.57	13	50S ribosomal subunit protein L15.
gi|16128162	P0A7V0	b0169	rpsB	S2	26.7	-25.03	15	30S ribosomal subunit protein S2.
gi|16131187	P62399	b3308	rplE	L5	20.3	-20.90	10	50S ribosomal subunit protein L5.
gi|16131186	P0AG59	b3307	rpsN	S14	11.6	-16.07	6	30S ribosomal subunit protein S14.
gi|16131176	P0A7R9	b3297	rpsK	S11	13.8	-13.87	8	30S ribosomal subunit protein S11.
gi|16131200	P0A7R5	b3321	rpsJ	S10	11.7	-12.17	7	30S ribosomal subunit protein S10.
gi|16132024	P0A7T7	b4202	rpsR	S18	9	-14.10	5	30S ribosomal subunit protein S18.
gi|16131813	P0A7J7	b3983	rplK	L11	14.9	-12.57	7	50S ribosomal subunit protein L11; in L1 translationally controlled operon with rplA (L1), rplJ, rplL, rpoB, rpoC
gi|16131196	P60422	b3317	rplB	L2	29.8	-8.80	5	50S ribosomal subunit protein L2.
gi|16131185	P0A7W7	b3306	rpsH	S8	14.1	-12.83	6	30S ribosomal subunit protein S8.
gi|290586581	P02358	b4200	rpsF	S6	15.7	-12.03	6	30S ribosomal subunit protein S6 .rpsF, 30S ribosomal protein S6
gi|16131195	P0A7U3	b3316	rpsS	S19	10.4	-12.33	6	30S ribosomal subunit protein S19.
gi|16131199	P60438	b3320	rplC	L3	22.2	-10.23	5	50S ribosomal subunit protein L3.
gi|16131192	P0ADY7	b3313	rplP	L16	15.3	-7.43	4	50S ribosomal subunit protein L16.
gi|16131076	P0AG48	b3186	rplU	L21	11.6	-6.20	4	50S ribosomal subunit protein L21; in operon with rpmA
gi|16131121	P0AA10	b3231	rplM	L13	16	-6.00	3	50S ribosomal subunit protein L13.
gi|16131075	P0A7L8	b3185	rpmA	L27	9.1	-5.80	1	50S ribosomal subunit protein L27; in operon with rplU
gi|16131815	P0A7J3	b3985	rplJ	L10	17.7	-5.67	4	50S ribosomal subunit protein L10.
gi|16128017	P0A7U7	b0023	rpsT	S20	9.7	-4.93	3	30S ribosomal subunit protein S20.
gi|16131173	P0AG44	b3294	rplQ	L17	14.4	-4.40	3	50S ribosomal subunit protein L17; in rpsD translationally controlled operon
gi|16129672	P0A7L3	b1716	rplT	L20	13.5	-4.40	2	50S ribosomal subunit protein L20.
gi|16131198	P60723	b3319	rplD	L4	22.1	-4.30	3	50S ribosomal subunit protein L4.
gi|16131183	P0C018	b3304	rplR	L18	12.8	-3.25	2	50S ribosomal subunit protein L18
gi|16131221	P0A7S3	b3342	rpsL	IsrA	13.7	-2.90	3	30S ribosomal subunit protein S12; in operon with rpsG (S7)
gi|16130527	P0A7K6	b2606	rplS	L19	13.1	-2.80	2	50S ribosomal subunit protein L19
gi|16131190	P0AG63	b3311	rpsQ	S17	9.7	-2.37	3	30S ribosomal subunit protein S17.
gi|16131057	P0ADZ4	b3165	rpsO	S15	10.3	-2.35	2	30S ribosomal subunit protein S15; in operon with truB and pnp
gi|16131188	P60624	b3309	rplX	L24	11.3	-2.30	1	50S ribosomal subunit protein L24.
gi|16131194	P68919	b3315	rplV	L22	12.2	-2.30	1	50S ribosomal subunit protein L22.
gi|16130961	P68679	b3065	rpsU	S21	8.5	-1.2	1	30S ribosomal subunit protein S21; in operon with dnaG and rpoD
gi|16128765	P25888	b0797	rhlE	RhlE	50	-3.60	1	ATP-dependent RNA helicase.
gi|16129170	P0A717	b1207	prs	Prs	34.2	-5.63	3	ribose-phosphate diphosphokinase (phosphoribosylpyrophosphate synthase)
gi|90111698	P21499	b4179	rnr	RNAseR	92.1	-4.83	2	exoribonuclease R; S1; aka VacB – can interact with DeaD
gi|16128848	P0A968	b0880	cspD	CspD	8	-3.20	4	inhibitor of DNA replication; cspD; S1
gi|16129047	P21513	b1084	rne	RNAseE	118.1	-5.80	2	ribonucleaseE: single strand endoribonuclease; component of RNA degradosome
gi|16131058	P60340	b3166	truB	TruB	35.1	-2.20	1	tRNA pseudouridine synthase B: tRNA pseudouridine(55) synthase and putative tmRNA pseudouridine(342) synthase
gi|16129633	P69776	b1677	lpp	Lpp	8.3	-3.90	1	murein lipoprotein; is one of the most abundant proteins in E. coli
gi|16129777	P0A9Y6	b1823	cspC	CspC	7.4	-2.30	2	member of the CspA-family.
gi|16130492	P0A7Y0	b2567	rnc	Rnc	25.5	-1.80	2	RNase III

GPM-265

rank	log(e)	log(I)	% (measured)	% (corrected)	unique	total	Mr	Accession	Description
1	-367.5	5.65	95	100+	32	140	11.2	gi|16131994|	global sRNA chaperone HF-I; host factor for RNA phage Q beta replication .
2	-358.9	4.68	55	63	31	49	61.1	gi|16128878|	30S ribosomal subunit protein S1 .
3	-288.2	5.2	64	84	27	49	25.9	gi|49176156|	RNA chaperone; probable regulator of ProP translation .
5	-148.1	3.68	12	15	15	15	150.5	gi|16131817|	RNA polymerase beta subunit .
7	-110.5	3.93	19	27	12	12	77.1	gi|145698316|	polynucleotide phosphorylase/polyadenylase .
8	-93	3.82	46	59	11	12	36.5	gi|16131174|	RNA polymerase alpha subunit .
9	-90.8	3.41	9.7	12	10	11	155.1	gi|16131818|	RNA polymerase beta prime subunit .
12	-81.5	4.06	48	63	8	11	20	gi|16131220|	30S ribosomal subunit protein S7 .
14	-79.1	3.48	16	21	9	9	70.5	gi|90111550|	ATP-dependent RNA helicase .
15	-72.4	4.12	77	92	8	19	6.9	gi|16130603|	pleiotropic regulatory protein for carbon source metabolism .
16	-70.1	3.9	38	46	7	10	24.7	gi|16131814|	50S ribosomal subunit protein L1 .
17	-64.9	3.74	26	30	6	7	36.8	gi|145698340|	tRNA-dihydrouridine synthase A; DusA is solely responsible for the 5,6-dihydrouridine modification observed in tRNA2fMet
18	-59.4	3.63	18	27	8	8	55.2	gi|16131602|	F1 sector of membrane-bound ATP synthase alpha subunit .
19	-49.7	4.06	47	68	6	8	13.1	gi|16131177|	30S ribosomal subunit protein S13 .
20	-48.8	3.72	28	40	7	8	23.5	gi|16131175|	30S ribosomal subunit protein S4 .
21	-46.8	3.6	32	35	4	4	15.8	gi|16132025|	50S ribosomal subunit protein L9 .
22	-45.1	3.69	40	45	5	6	17.6	gi|16131182|	30S ribosomal subunit protein S5 .
23	-45	4.02	32	46	6	6	14.8	gi|16131120|	30S ribosomal subunit protein S9 .
24	-42.2	3.71	21	29	6	6	26	gi|16131193|	30S ribosomal subunit protein S3 .
25	-39.1	3.58	29	35	5	8	26.7	gi|16128162|	30S ribosomal subunit protein S2 .
26	-36.8	3.22	47	77	4	4	11.6	gi|16131186|	30S ribosomal subunit protein S14 .
27	-34.4	3.38	5.7	8	4	5	87.4	gi|16128424|	DNA-binding ATP-dependent protease La .
28	-34.2	3.54	29	38	4	5	20.3	gi|16131187|	50S ribosomal subunit protein L5 .
29	-34.1	3.46	32	43	4	4	15	gi|16131180|	50S ribosomal subunit protein L15 .
30	-24.5	3.13	33	42	4	4	13.8	gi|16131176|	30S ribosomal subunit protein S11 .
31	-21.3	2.78	5.2	7	3	3	85.1	gi|90111588|	NP_417866; yhgF; has C-terminal S1
32	-18.3	2.85	17	30	2	2	16.7	gi|16131143|	acetyl CoA carboxylase; BCCP subunit .
34	-17.6	3.18	14	17	2	2	14.1	gi|16131185|	30S ribosomal subunit protein S8 .
35	-15.8	2.47	8.4	10	3	3	55.3	gi|90111563|	ribonuclease G .
36	-14.6	2.38	38	43	1	1	4.8	gi|49176463|	entericidin B membrane lipoprotein .
37	-13.5	2.66	23	47	1	1	9	gi|16132024|	30S ribosomal subunit protein S18 .
38	-12.1	3.2	7.5	10	3	3	53.8	gi|162135892|	poly(A) polymerase .
39	-10.3	2.98	12	13	2	2	14.9	gi|16131813|	50S ribosomal subunit protein L11 .
40	-9.6	3.03	12	14	2	2	15.7	gi|290586581|	rpsF: 30S ribosomal subunit protein S6 .
41	-8.1	3.57	23	32	2	2	10.4	gi|16131195|	30S ribosomal subunit protein S19 .
42	-7.5	2.88	7.3	10	1	1	17.7	gi|16131815|	50S ribosomal subunit protein L10 .
43	-6.3	2.59	3.5	5	1	1	34.2	gi|16129170|	phosphoribosylpyrophosphate synthase .
44	-6.2	2.64	18	31	2	2	14.4	gi|16131173|	50S ribosomal subunit protein L17 .
45	-5.8	2.82	9.6	18	1	1	15.3	gi|16131192|	50S ribosomal subunit protein L16 .
46	-5	2.46	11	23	1	1	9.7	gi|16128017|	30S ribosomal subunit protein S20 .
47	-5	2.14	1.2	2	1	1	92.1	gi|90111698|	exoribonuclease R; S1-domain; RNase R .
48	-4.2	2.9	22	25	1	1	8	gi|16128848|	inhibitor of DNA replication; cspD; S1-domain;cold shock protein homolog .
49	-4	2.68	12	17	1	1	13.1	gi|16130527|	rplS: 50S ribosomal subunit protein L19
50	-3.2	2.38	4.3	5	1	1	22.2	gi|16131199|	50S ribosomal subunit protein L3 .
51	-3	2.3	5.6	8	1	1	13.7	gi|16131221|	30S ribosomal subunit protein S12 .
52	-2.8	2.46	6.8	11	1	1	12.8	gi|16131183|	rplR: 50S ribosomal subunit protein L18
53	-2.5	2.62	6.3	9	1	1	16	gi|16131121|	50S ribosomal subunit protein L13 .
54	-2.3	1.76	7	12	1	1	22.1	gi|16131198|	50S ribosomal subunit protein L4 .
55	-2.1	2.38	7.7	11	1	1	29.8	gi|16131196|	50S ribosomal subunit protein L2 .
56	-2.1	3.27	8.7	12	1	1	11.7	gi|16131200|	30S ribosomal subunit protein S10 .
57	-1.9	2.75	8.3	15	1	1	9.7	gi|16131190|	30S ribosomal subunit protein S17 .
58	-1.3	3.09	11	19	1	1	18	gi|16128042|	folA: dihydrofolate reductase type I; trimethoprim resistance
59	-1.3	1.55	3.3	4	1	1	42.9	gi|16131655|:reversed	no protein information available
60	-1.2	1.99	1.5	2	1	1	87.4	gi|16129658|	phosphoenolpyruvate synthase [Escherichia coli K12].
61	-1.2	2.53	2.9	3	1	1	42.5	gi|16129756|:reversed	no protein information available
62	-1.1	2.55	3.5	4	1	1	25.5	gi|16130492|	RNase III .

GPM-264

rank	log(e)	log(I)	% (measured)	% (corrected)	unique	total	Mr	Accession	Description
1	-215.3	4.12	36	42	19	28	61.1	gi|16128878|	30S ribosomal subunit protein S1 .
2	-206.4	5.02	66	100+	18	89	11.2	gi|16131994|	global sRNA chaperone HF-I
3	-193.8	4.58	53	69	19	37	25.9	gi|49176156|	RNA chaperone; probable regulator of ProP translation .
5	-111.2	3.89	23	32	13	14	77.1	gi|145698316|	polynucleotide phosphorylase/polyadenylase .
6	-101.3	3.71	8.5	10	11	11	150.5	gi|16131817|	RNA polymerase beta subunit .
9	-69	3.43	12	16	7	7	70.5	gi|90111550|	ATP-dependent RNA helicase .
10	-64.4	4	31	41	8	9	20	gi|16131220|	30S ribosomal subunit protein S7 .
11	-59.5	3.3	13	19	6	6	55.2	gi|16131602|	F1 sector of membrane-bound ATP synthase alpha subunit .
12	-58	4.14	28	34	5	9	24.7	gi|16131814|	50S ribosomal subunit protein L1 .
14	-45.5	3.35	21	28	5	6	26	gi|16131193|	30S ribosomal subunit protein S3 .
16	-43.5	3.42	20	26	7	7	36.5	gi|16131174|	RNA polymerase alpha subunit .
17	-42.1	3.52	37	40	5	5	15.8	gi|16132025|	50S ribosomal subunit protein L9 .
18	-41.5	4.11	21	30	5	9	23.5	gi|16131175|	30S ribosomal subunit protein S4 .
19	-38.5	3.1	40	46	6	7	17.6	gi|16131182|	30S ribosomal subunit protein S5 .
20	-37.9	3.51	38	52	5	5	15	gi|16131180|	50S ribosomal subunit protein L15 .
21	-33.9	3.77	30	43	4	5	13.1	gi|16131177|	30S ribosomal subunit protein S13 .
22	-33.2	3.66	25	37	4	5	14.8	gi|16131120|	30S ribosomal subunit protein S9 .
24	-24.2	3.02	5.5	8	4	4	87.4	gi|16128424|	DNA-binding ATP-dependent protease La .
25	-23.9	3.79	48	57	3	4	6.9	gi|16130603|	pleiotropic regulatory protein for carbon source metabolism .
26	-23.4	2.43	2.7	3	3	3	155.1	gi|16131818|	RNA polymerase beta prime subunit .
27	-21.2	3.28	29	39	3	3	11.7	gi|16131200|	30S ribosomal subunit protein S10 .
28	-18	3.04	21	24	3	3	14.9	gi|16131813|	50S ribosomal subunit protein L11 .
29	-17.8	3	15	22	3	3	29.8	gi|16131196|	50S ribosomal subunit protein L2 .
30	-14.3	3.12	24	31	3	3	13.8	gi|16131176|	30S ribosomal subunit protein S11 .
31	-13.8	3.36	11	12	3	3	36.8	gi|145698340|	tRNA-dihydrouridine synthase A .
32	-13.4	3.66	23	32	2	2	10.4	gi|16131195|	30S ribosomal subunit protein S19 .
33	-12.3	3.07	6	8	3	3	53.8	gi|162135892|	poly(A) polymerase .
34	-12.3	3.23	12	14	2	2	15.7	gi|290586581|	rpsF: 30S ribosomal subunit protein S6 .
35	-12.1	2.32	38	43	1	1	4.8	gi|49176463|	entericidin B membrane lipoprotein .
36	-11.5	2.48	21	39	2	2	15.3	gi|16131192|	50S ribosomal subunit protein L16 .
37	-11.3	2.77	7.7	9	2	2	22.2	gi|16131199|	50S ribosomal subunit protein L3 .
38	-9.5	2.73	13	17	2	2	16	gi|16131121|	50S ribosomal subunit protein L13 .
39	-9.5	2.53	14	17	2	2	14.1	gi|16131185|	30S ribosomal subunit protein S8 .
40	-8.8	2.09	7	12	1	1	22.1	gi|16131198|	50S ribosomal subunit protein L4 .
41	-7.3	2.28	16	22	2	2	17.7	gi|16131815|	50S ribosomal subunit protein L10 .
42	-6.4	2.32	11	23	1	1	9.7	gi|16128017|	30S ribosomal subunit protein S20 .
43	-6.2	2.95	19	25	2	2	11.6	gi|16131076|	50S ribosomal subunit protein L21 .
44	-5.9	2.28	1.2	2	1	1	92.1	gi|90111698|	exoribonuclease R; RNase R .
45	-5.6	2.41	3.3	4	1	1	55.3	gi|90111563|	ribonuclease G .
46	-5	3.16	5.9	9	1	1	13.5	gi|16129672|	50S ribosomal subunit protein L20 .
48	-4.8	2.19	7.1	8	1	1	26.7	gi|16128162|	30S ribosomal subunit protein S2 .
49	-4.7	2.55	8.9	15	1	1	11.6	gi|16131186|	30S ribosomal subunit protein S14 .
50	-4.7	2.54	5.1	9	1	1	16.7	gi|16131143|	acetyl CoA carboxylase; BCCP subunit .
51	-3.9	1.74	17	21	1	1	8.3	gi|16129633|	murein lipoprotein .
52	-3.6	1.97	2.4	3	1	1	50	gi|16128765|	ATP-dependent RNA helicase .
53	-3.3	2.43	3.8	5	1	1	34.2	gi|16129170|	phosphoribosylpyrophosphate synthase .
54	-3.2	2.21	15	31	1	1	9	gi|16132024|	
55	-2.6	3.17	5.6	8	1	1	13.7	gi|16131221|	30S ribosomal subunit protein S12 .
56	-2.6	2.55	7.9	14	1	1	14.4	gi|16131173|	50S ribosomal subunit protein L17 .
57	-2.6	2.93	8.3	15	1	1	9.7	gi|16131190|	30S ribosomal subunit protein S17 .
58	-2.6	2.45	6.7	9	1	1	10.3	gi|16131057|	30S ribosomal subunit protein S15 .
59	-2.5	2.21	3.1	4	1	1	25.5	gi|16130492|	RNase III .
60	-2.3	2.34	8.7	13	1	1	11.3	gi|16131188|	50S ribosomal subunit protein L24 .
61	-2.3	2.42	22	25	1	2	8	gi|16128848|	inhibitor of DNA replication; cold shock protein homolog .
62	-2.2	2.6	3.2	4	1	1	35.1	gi|16131058|	tRNA pseudouridine synthase B: tRNA pseudouridine(55) synthase and putative tmRNA pseudouridine(342) synthase
63	-2	3.11	3.9	5	1	1	20.3	gi|16131187|	50S ribosomal subunit protein L5 .
64	-1.6	2.68	7.4	9	1	1	14.9	gi|16129609|:reversed	no protein information available
65	-1.4	2.55	3.2	4	1	1	32.4	gi|171701684|	DNA-binding transcriptional activator for rhaSR; L-rhamnose-binding .
66	-1.2	3.04	9.9	25	1	1	8.5	gi|16130961|	30S ribosomal subunit protein S21 .
67	-1.2	3.29	1.8	2	1	1	42.4	gi|16129802|:reversed	no protein information available
68	-1.1	3.01	16	18	1	1	7.4	gi|16129777|	stress protein; member of the CspA-family .
69	-1.1	2.19	1.6	2	1	1	52.8	gi|90111464|	phosphatidylserine synthase (CDP-diacylglycerol-serine O-phosphatidyltransferase)

GPM-266

rank	log(e)	log(I)	% (measured)	% (corrected)	unique	total	Mr	Accession	Description
1	-325.3	4.55	48	54	28	48	61.1	gi|16128878|	30S ribosomal subunit protein S1 .
2	-315.9	5.51	94	100+	27	133	11.2	gi|16131994|	global sRNA chaperone HF-I
3	-232.7	4.91	59	77	22	42	25.9	gi|49176156|	RNA chaperone; probable regulator of ProP translation .
5	-168.6	3.91	30	42	18	18	77.1	gi|145698316|	polynucleotide phosphorylase/polyadenylase .
9	-80.1	3.35	22	32	9	9	55.2	gi|16131602|	F1 sector of membrane-bound ATP synthase alpha subunit .
10	-74.8	4.55	79	94	9	15	6.9	gi|16130603|	pleiotropic regulatory protein for carbon source metabolism .
11	-73.8	4.01	37	48	9	10	20	gi|16131220|	30S ribosomal subunit protein S7 .
12	-69.6	3.38	5.7	7	7	7	150.5	gi|16131817|	RNA polymerase beta subunit .
14	-65	3.45	9.2	13	8	8	87.4	gi|16128424|	DNA-binding ATP-dependent protease La .
15	-64.1	3.25	28	36	9	9	36.5	gi|16131174|	RNA polymerase alpha subunit .
16	-63	3.35	26	36	9	10	26	gi|16131193|	30S ribosomal subunit protein S3
17	-58.6	3.83	26	32	5	7	24.7	gi|16131814|	50S ribosomal subunit protein L1 .
18	-49.5	3.4	26	30	6	6	36.8	gi|145698340|	tRNA-dihydrouridine synthase A .
19	-49.3	3.22	12	15	6	6	70.5	gi|90111550|	ATP-dependent RNA helicase .
20	-42.3	3.85	32	46	6	8	14.8	gi|16131120|	30S ribosomal subunit protein S9 .
21	-39.2	3	4.8	6	5	5	155.1	gi|16131818|	RNA polymerase beta prime subunit .
22	-39.1	3.02	34	39	4	4	17.6	gi|16131182|	30S ribosomal subunit protein S5 .
23	-38.8	3.81	37	54	4	7	13.1	gi|16131177|	30S ribosomal subunit protein S13 .
24	-34.7	3.35	31	42	4	4	15	gi|16131180|	50S ribosomal subunit protein L15 .
25	-31.2	3.59	19	23	5	6	26.7	gi|16128162|	30S ribosomal subunit protein S2 .
26	-27.9	3.61	17	25	4	7	23.5	gi|16131175|	30S ribosomal subunit protein S4 .
27	-26.5	3.16	29	38	4	4	20.3	gi|16131187|	50S ribosomal subunit protein L5 .
28	-25.6	3.45	37	78	2	3	9	gi|16132024|	30S ribosomal subunit protein S18 .
30	-19.6	3.47	14	15	2	2	15.8	gi|16132025|	50S ribosomal subunit protein L9 .
31	-17.4	2.68	9.2	11	3	3	55.3	gi|90111563|	ribonuclease G .
32	-16.2	2.74	13	15	2	2	22.2	gi|16131199|	50S ribosomal subunit protein L3 .
33	-15.5	3.59	23	32	2	2	10.4	gi|16131195|	30S ribosomal subunit protein S19 .
34	-14.2	3.19	12	14	2	2	15.7	gi|290586581|	rpsF: 30S ribosomal subunit protein S6
35	-13.2	3.1	26	35	3	3	11.7	gi|16131200|	30S ribosomal subunit protein S10 .
36	-12.8	3.31	6	8	3	3	53.8	gi|162135892|	poly(A) polymerase .
37	-11.4	2.81	14	17	2	2	14.1	gi|16131185|	30S ribosomal subunit protein S8 .
38	-10.5	1.97	38	43	1	1	4.8	gi|49176463|	entericidin B membrane lipoprotein .
39	-9.4	2.96	12	13	2	2	14.9	gi|16131813|	50S ribosomal subunit protein L11 .
40	-7.3	2.83	3.5	5	1	1	34.2	gi|16129170|	phosphoribosylpyrophosphate synthase .
41	-6.7	2.37	18	30	1	1	11.6	gi|16131186|	30S ribosomal subunit protein S14 .
43	-6.5	2.43	7.7	11	1	1	29.8	gi|16131196|	50S ribosomal subunit protein L2 .
44	-6.2	2.86	19	25	2	2	11.6	gi|16131076|	50S ribosomal subunit protein L21 .
45	-5.8	2.43	15	27	1	1	9.1	gi|16131075|	rpmA: 50S ribosomal subunit protein L27
46	-5.8	3.69	2.5	4	2	2	118.1	gi|16129047|	fused ribonucleaseE: endoribonuclease/RNA-binding protein/RNA degradosome binding protein
47	-5	2.36	9.6	18	1	1	15.3	gi|16131192|	50S ribosomal subunit protein L16 .
48	-4.7	2.7	5.1	9	1	1	16.7	gi|16131143|	acetyl CoA carboxylase; BCCP subunit .
49	-3.8	3.09	5.9	9	1	1	13.5	gi|16129672|	50S ribosomal subunit protein L20 .
50	-3.7	2.15	6.8	11	1	1	12.8	gi|16131183|	rplR: 50S ribosomal subunit protein L18
51	-3.6	2.15	1.2	2	1	1	92.1	gi|90111698|	exoribonuclease R; RNase R .
52	-3.4	1.79	11	23	1	1	9.7	gi|16128017|	30S ribosomal subunit protein S20 .
53	-3.1	3.05	22	25	1	1	8	gi|16128848|	inhibitor of DNA replication; cold shock protein homolog .
54	-3.1	2.93	5.6	8	1	1	13.7	gi|16131221|	30S ribosomal subunit protein S12 .
55	-2.8	1.41	8.5	11	1	1	13.8	gi|16131176|	30S ribosomal subunit protein S11 .
56	-2.6	2.96	8.3	15	1	1	9.7	gi|16131190|	30S ribosomal subunit protein S17 .
57	-2.5	1.66	1.6	2	1	1	85.1	gi|90111588|	predicted transcriptional accessory protein .
58	-2.3	2.73	16	18	1	1	7.4	gi|16129777|	stress protein; member of the CspA-family .
59	-2.3	2.33	8.2	12	1	1	12.2	gi|16131194|	rplV: 50S ribosomal subunit protein L22
60	-2.2	2.21	0.9	1	1	1	97.3	gi|16129235|	DNA topoisomerase I omega subunit .
61	-2.2	1.85	7.3	10	1	1	17.7	gi|16131815|	50S ribosomal subunit protein L10 .
62	-2.1	1.89	6.7	9	1	1	10.3	gi|16131057|	30S ribosomal subunit protein S15 .
63	-1.8	1.71	7	12	1	1	22.1	gi|16131198|	50S ribosomal subunit protein L4 .
64	-1.6	2.54	12	17	1	1	13.1	gi|16130527|	rplS: 50S ribosomal subunit protein L19
66	-1.2	1.42	4.7	6	1	1	24.3	gi|16129027|:reversed	no protein information available
67	-1	2.48	3.2	4	1	1	32.4	gi|171701684|	DNA-binding transcriptional activator for rhaSR; L-rhamnose-binding.
